# Brain-Targeted Biomimetic Nanodecoys with Neuroprotective Effects for Precise Therapy of Parkinson’s Disease

**DOI:** 10.1021/acscentsci.2c00741

**Published:** 2022-09-13

**Authors:** Yao Liu, Jingshan Luo, Yujing Liu, Wen Liu, Guangtao Yu, Yuting Huang, Yu Yang, Xiaojia Chen, Tongkai Chen

**Affiliations:** † Science and Technology Innovation Center, Guangzhou University of Chinese Medicine, Guangzhou 510405, China; ‡ State Key Laboratory of Quality Research in Chinese Medicine, Institute of Chinese Medical Sciences, University of Macau, Macau 999078, China; § Stomatological Hospital, Southern Medical University, Guangzhou 510280, China; ∥ Institute of Molecular Medicine (IMM), Renji Hospital, School of Medicine, Shanghai Jiao Tong University, Shanghai 200240, China

## Abstract

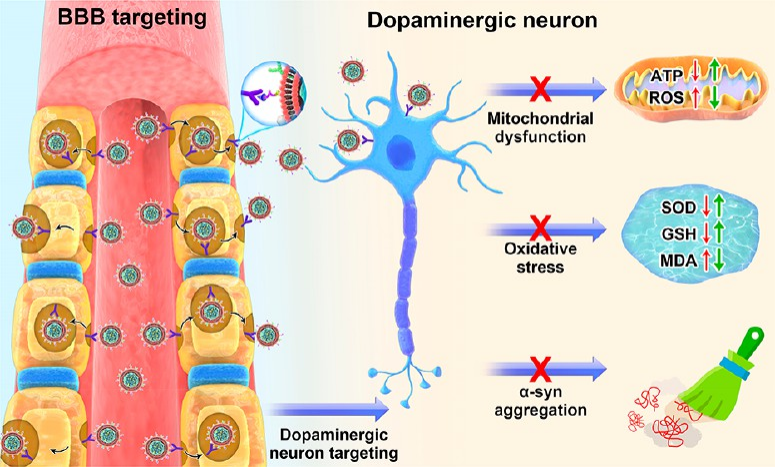

Parkinson’s disease (PD) is a neurodegenerative disorder characterized by the gradual loss of dopaminergic neurons in the substantia nigra and the accumulation of α-synuclein aggregates called Lewy bodies. Here, nanodecoys were designed from a rabies virus polypeptide with a 29 amino acid (RVG29)-modified red blood cell membrane (RBCm) to encapsulate curcumin nanocrystals (Cur-NCs), which could effectively protect dopaminergic neurons. The RVG29-RBCm/Cur-NCs nanodecoys effectively escaped from reticuloendothelial system (RES) uptake, enabled prolonged blood circulation, and enhanced blood–brain barrier (BBB) crossing after systemic administration. Cur-NCs loaded inside the nanodecoys exhibited the recovery of dopamine levels, inhibition of α-synuclein aggregation, and reversal of mitochondrial dysfunction in PD mice. These findings indicate the promising potential of biomimetic nanodecoys in treating PD and other neurodegenerative diseases.

## Abstract 2

Novel biomimetic nanodecoys derived from a RVG29-modified red blood cell membrane with curcumin nanocrystals encapsulated are presented, which exhibited efficient BBB permeability and excellent anti-Parkinsonian efficacy.

## Introduction

Parkinson’s disease (PD) is the second most common neurodegenerative disease in middle-aged and older individuals.^1^ Its main pathological manifestations are the degeneration and necrosis of dopaminergic (DA) neurons in the substantia nigra and the development of Lewy bodies primarily consisting of misfolded α-synuclein (α-syn).^2^ Currently, drug treatment for PD is focused on levodopa (l-DOPA), which alleviates clinical symptoms but does not delay PD development. With PD progression, the required dosage and frequency of l-DOPA administration increase.^3^ Therefore, there is an urgent need to develop new strategies to improve the specificity and efficacy of PD therapy.

Curcumin (Cur), a natural product, has a wide range of pharmacological effects, including anti-inflammatory, antioxidant, immune regulation, and apoptosis regulation effects.^4−6^ Cur also shows good therapeutic activity against PD and can inhibit α-syn aggregation.^7,8^ However, the greatest obstacles hindering Cur application are systemic cytotoxicity and rapid clearance due to poor drug selectivity.^9^ An ideal Cur delivery system for PD therapy should target the brain and allow the accumulation of the therapeutic drug at the site of action.^10^ Such a system would need to overcome the inherent challenges such as limited blood circulation (e.g., reticuloendothelial system (RES) uptake), the blood–brain barrier (BBB), and brain-targeted delivery.^11−13^

In the recent decade, red blood cell membrane (RBCm)-coated nanodecoys have shown a superprolonged systemic retention time (*T*_1/2_ = 39.6 h in mice) by reducing immune recognition and reticuloendothelial system (RES) uptake.^14^ Cell membrane-coated nanomedicines have shown great potential for enhanced cancer therapy and toxin neutralization due to prolonged blood circulation,^15−19^ whereas employing RBCm-based biomimetic nanodecoys for treatment of neurodegenerative diseases is also challenged due to the blood–brain barrier (BBB) and site-specific delivery to nerve cells. Engineering RBC membranes with targeting ligands that simultaneously cross the BBB and bind to nerve cells may possibly enhance drug accumulation for PD treatment.

**Figure 1:**
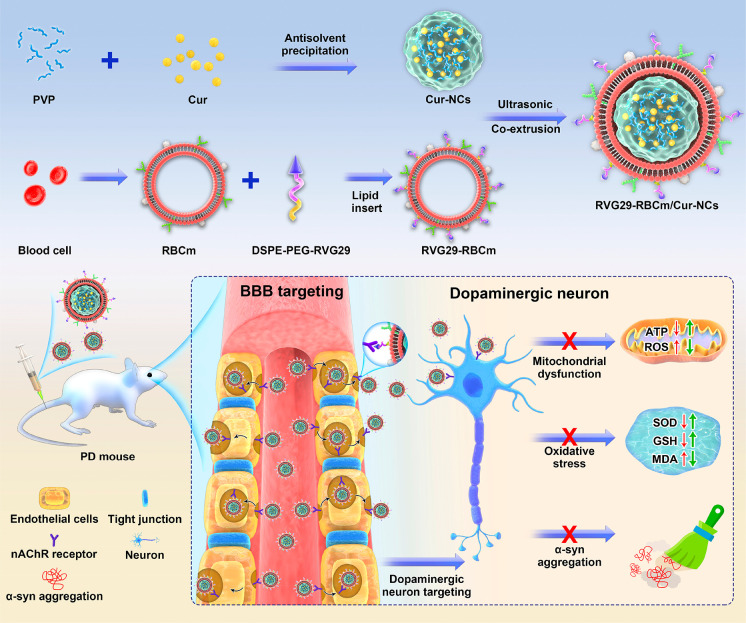
Schematic overview illustrating the preparation and application of RVG29-RBCm/Cur-NCs for PD therapy.

Herein, we developed a brain-targeted biomimetic nanodecoy for the treatment of PD (Figure 1). In this biomimetic system, the natural RBCm was used as the drug carrier and modified with the brain-targeting peptide RVG29 (RVG29-RBCm), which is a rabies virus polypeptide with 29 amino acids (RVG29) that can specifically bind to acetylcholine receptors (nAChR) expressed in both the BBB and neuronal cells.^20,21^ Subsequently, Cur-based drug nanocrystals (Cur-NCs), pure particles of the drug,^22,23^ were loaded into the nanodecoys to generate brain-targeted biomimetic Cur-NCs (RVG29-RBCm/Cur-NCs), showing extremely high drug loading yields. After systemic administration, such RVG29-RBCm/Cur-NCs nanodecoys overcome the problems of limited blood circulation, low BBB penetration, and poor neuronal targeting and thereby achieve efficient drug delivery to the brain. Importantly, the in vivo results demonstrated that RVG29-RBCm/Cur-NCs could ameliorate motor deficits in PD mouse models, reduce the loss of tyrosine hydroxylase-positive (TH^+^) neurons in the substantia nigra compacta (SNpc), and restore DA levels in the striatum. Further exploration showed that the therapeutic effects of RVG29-RBCm/Cur-NCs were achieved via the inhibition of abnormal α-syn aggregation, promotion of TH expression, antioxidative effects, and reversal of mitochondrial dysfunction. Together, the findings demonstrated the promising neuroprotective effects of RVG29-RBCm/Cur-NCs nanodecoys in PD therapy.

## Results and Discussion

### Preparation and Characterization of Cur-NCs

**Figure 2:**
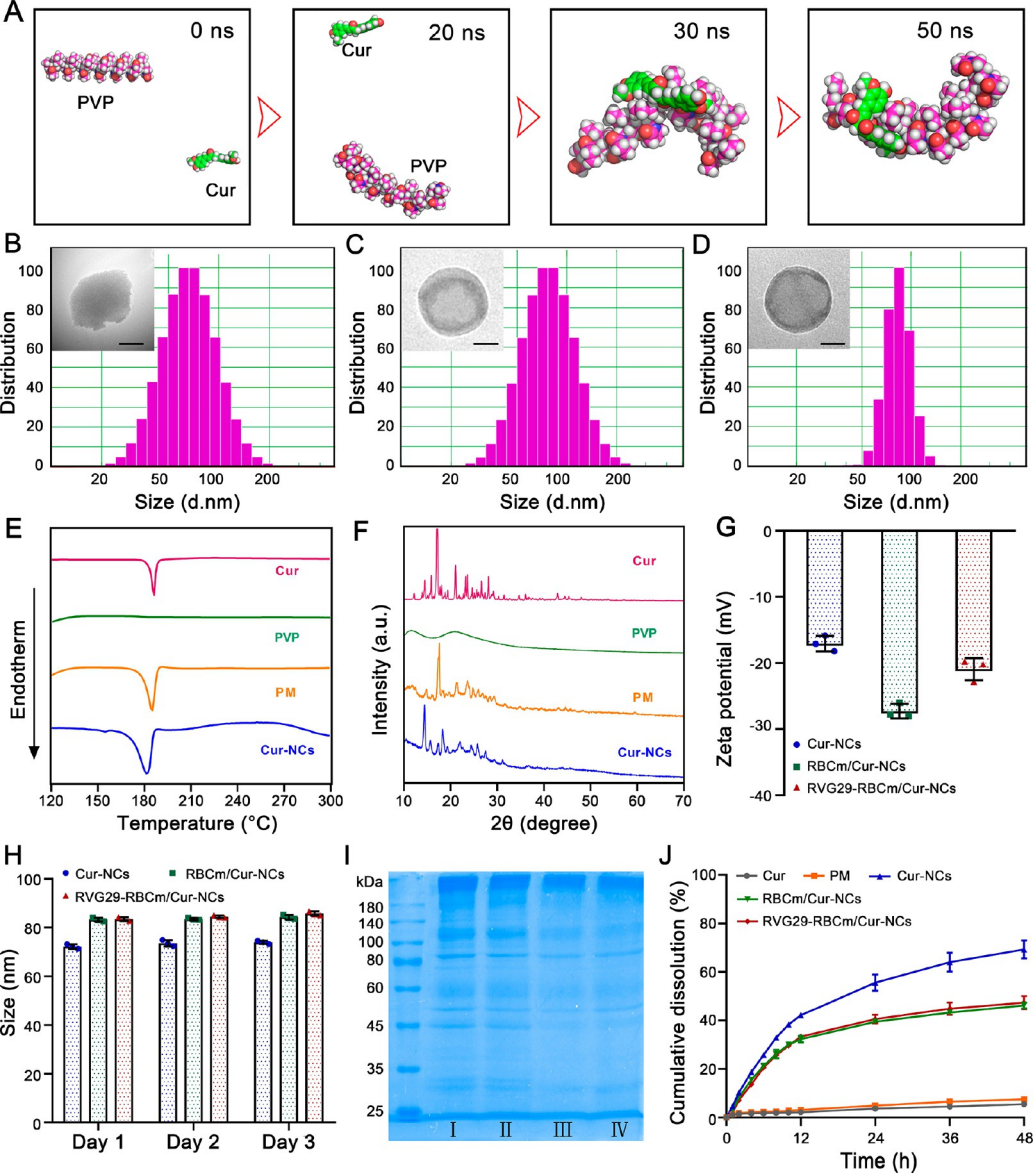
Characterization of Cur-NCs, RBCm/Cur-NCs, and RVG29-RBCm/Cur-NCs. (A) Molecular dynamics simulation of Cur and PVP at 0, 20, 30, and 50 ns. (B–D) Particle size distributions and transmission electron microscopy (TEM) images of (B) Cur-NCs, (C) RBCm/Cur-NCs, and (D) RVG29-RBCm/Cur-NCs. Scale bar: 25 nm. (E) DSC curves of Cur-NCs. (F) XRD patterns of Cur-NCs. (G) Zeta potential of Cur-NCs, RBCm/Cur-NCs, and RVG29-RBCm/Cur-NCs (*n* = 3). (H) Stability investigation (*n* = 3). (I) SDS-PAGE evaluation of RBCm (I), RVG29-RBCm (II), RBCm/Cur-NCs (III), and RVG29-RBCm/Cur-NCs (IV). (J) In vitro drug release from different Cur formulations (*n* = 3).

To prepare Cur-NCs, a series of stabilizers and concentrations was examined. After screening, a 0.8 mg/mL aqueous solution of Cur-NCs in polyvinylpyrrolidone K90 (PVP K90, the preferred stabilizer for inhibiting particle aggregation) was found to be suitable for further preparation (Figure S1). Molecular dynamics simulation was performed to examine the binding and interaction between Cur and PVP. As shown in Figure 2A, PVP and Cur molecules gradually approached each other and interacted during a 50 ns molecular dynamics progression from a disordered random distribution state. In the kinetic process, van der Waals and electrostatic forces decreased after 20 ns and the spatial distance decreased gradually (Figure S2). The interaction energy between PVP and Cur molecules in the system gradually decreased and tended to become stable, indicating that the two molecules reached an equilibrium state through interaction in the aqueous solution (Figure S3). After the 20–50 ns window, the energy remained in equilibrium and a stable state. Calculations showed that the binding free energy (Δ*G*_total_) between PVP and Cur was −27.601 kJ/mol. The contributions of van der Waals forces and electrostatic interaction to Δ*G*_total_, expressed as Δ*E*_vdw_ and Δ*E*_elec_, were −49.102 and −7.339 kJ/mol, respectively. Regarding the solvent effect, the contribution of the polar part to the free energy was 35.013 kJ/mol and that of the nonpolar part was −6.174 kJ/mol. These results revealed that the van der Waals force played a major role in the spontaneous combination of PVP and Cur.

The average particle size of optimized Cur-NCs was 71.3 nm, and they had an irregular spherical shape (Figure 2B). Differential scanning calorimetry (DSC) curves of the Cur powder, PVP K90 powder, PM (a physical mixture of Cur and PVP K90), and Cur-NCs showed a similar single Cur endothermic peak near 180 °C (Figure 2E). This indicated that the Cur-NCs were crystalline. X-ray diffraction (XRD) spectra of Cur-NCs showed a peak characteristic of Cur (Figure 2F), suggesting that the crystalline state of Cur remained unchanged during antisolvent precipitation.

### Preparation and Characterization of RVG29-RBCm/Cur-NCs

The successful synthesis of DSPE-PEG_2000_-RVG29 was evidenced by the band coincidence in the ^1^H NMR spectra (Figure S4). In DSPE-PEG_2000_, the characteristic methylene absorption peak of PEG appeared at 3.50 ppm and the −CH_2_– peak of DSPE appeared at 1.25 ppm. The RCONH– peak of RVG29 was observed at 2.10 ppm, and the characteristic peaks of the aromatic protons present in RVG29 amino acids could also be observed at 6.50–8.50 ppm.

Next, DSPE-PEG_2000_-RVG29 was further modified using the RBCm through lipid insertion to achieve the brain-targeting functionalization. The derived RBCm was coated onto Cur-NCs through ultrasonic treatment followed by coextrusion using an Avanti miniextruder. Proteins in the serum bind to the surface of nanoparticles, and the cross-linking between nanoparticles was achieved via interaction between proteins, resulting in absorbance changes.^14^ Short-term changes in the absorbance values of serum, RBCm, Cur-NCs, and RBCm/Cur-NCs were measured at 560 nm using different RBCm:Cur-NCs ratios (Figure S5). Compared with Cur-NCs, RBCm/Cur-NCs showed lower changes in absorbance values, consistent with the expectation that the RBCm can improve Cur-NC stability. For RBCm/Cur-NCs, the best stability was observed when the RBCm:Cur-NCs ratio was 1:4. Therefore, this volume ratio was used for the preparation of biomimetic NCs.

Due to coating with the RBCm, the surface of RBCm/Cur-NCs had an obvious “core–shell” structure. In addition, repeated extrusion during the preparation process made these NCs more spherical (Figure 2C and Figure S6), and their particle size increased to 82.7 nm. RVG29-RBCm/Cur-NCs had a similar particle size (84.3 nm) and morphology, and the particle size distribution was more concentrated (Figure 2D and Figure S7). The zeta potential of Cur-NCs was −17.07 mV. After coating with the RBCm, the potential decreased to −27.29 mV. After modification with the positively charged peptide RVG29 (RVG29-RBCm/Cur-NCs), the zeta potential increased to −20.94 mV (Figure 2G). Given that the nanoparticles had negative charges, we speculated that they would not be nonspecifically uptaken by cells through charge-based binding during circulation.^24^ Cur, Cur-NCs, RBCm/Cur-NCs, and RVG29-RBCm/Cur-NCs showed similar characteristic absorption peaks at 427 nm (Figure S8), demonstrating that Cur was efficiently loaded onto Cur-NCs, RBCm/Cur-NCs, and RVG29-RBCm/Cur-NCs. The drug loading capacity of RVG29-RBCm/Cur-NCs was 5.13 ± 0.31%, and the encapsulation efficiency was 99.47 ± 0.11%. During a 3-day stability examination, the particle sizes of Cur-NCs, RBCm/Cur-NCs, and RVG29-RBCm/Cur-NCs did not change significantly, indicating that they had good stability (Figure 2H).

After determining the physical characteristics of RVG29-RBCm/Cur-NCs, their biological characteristics were evaluated. RBCm, RVG29-RBCm, RBCm/Cur-NCs, and RVG29-RBCm/Cur-NCs had similar protein compositions of RBCm, indicating successful RBCm coating on Cur-NCs (Figure 2I). These biomimetic nanodecoys possessed the properties of RBCm, which was beneficial for Cur-NCs to realize immune evasion during circulation in vivo.

Finally, the in vitro release behavior was examined to investigate the drug release properties of the prepared Cur formulations (Figure 2J). The solubility of Cur (suspended in PBS) was very low, and the release was less than 6% within 48 h. The physical mixture of Cur and PVP K90 (indicated as “PM”) showed a release of only 7.49%, demonstrating that PVP K90 could not improve the release behavior of Cur. The cumulative release of Cur from Cur-NCs within 48 h was increased to 69.25%, suggesting that the formation of nanocrystals improved the solubility of Cur and provided rapid release. Notably, the cumulative release observed in RBCm/Cur-NCs and RVG29-RBCm/Cur-NCs was 46.20% and 47.37%, respectively, indicating a good sustained-release effect with an improvement in Cur solubility. These results demonstrated that coating with the RBCm increased the stability of Cur-NCs and allowed controlled drug release.

### Immune Evasion

The RBCm has been reported to show features such as being “stealth” and can prevent recognition and clearance by macrophages in vivo. Therefore, RAW264.7 cells were chosen to study the immune evasion ability of biomimetic Cur nanopreparations. Since Cur itself has fluorescence properties (Ex/Em: 425/530 nm), the internalization of Cur preparations could be evaluated based on spontaneous fluorescence. The coincubation of RAW264.7 cells with a Cur nanopreparation did not cause any cytotoxicity (Figure S9). Confocal laser scanning microscopy (CLSM) revealed a weak fluorescence signal in the RBCm/Cur-NCs and RVG29-RBCm/Cur-NCs groups, although a higher fluorescence intensity was observed in the Cur-NCs group (Figure S10). This reduction in Cur uptake demonstrated the reduced targeting of RBCm/Cur-NCs and RVG29-RBCm/Cur-NCs by macrophages. This could be because the RBCm expressing CD47 proteins can send a “don’t eat me” signal to macrophages, thereby preventing the nonspecific uptake of coated particles by macrophages, which could prolong the circulation of drugs in vivo.^14^

### Transport Across an In Vitro BBB Model

**Figure 3:**
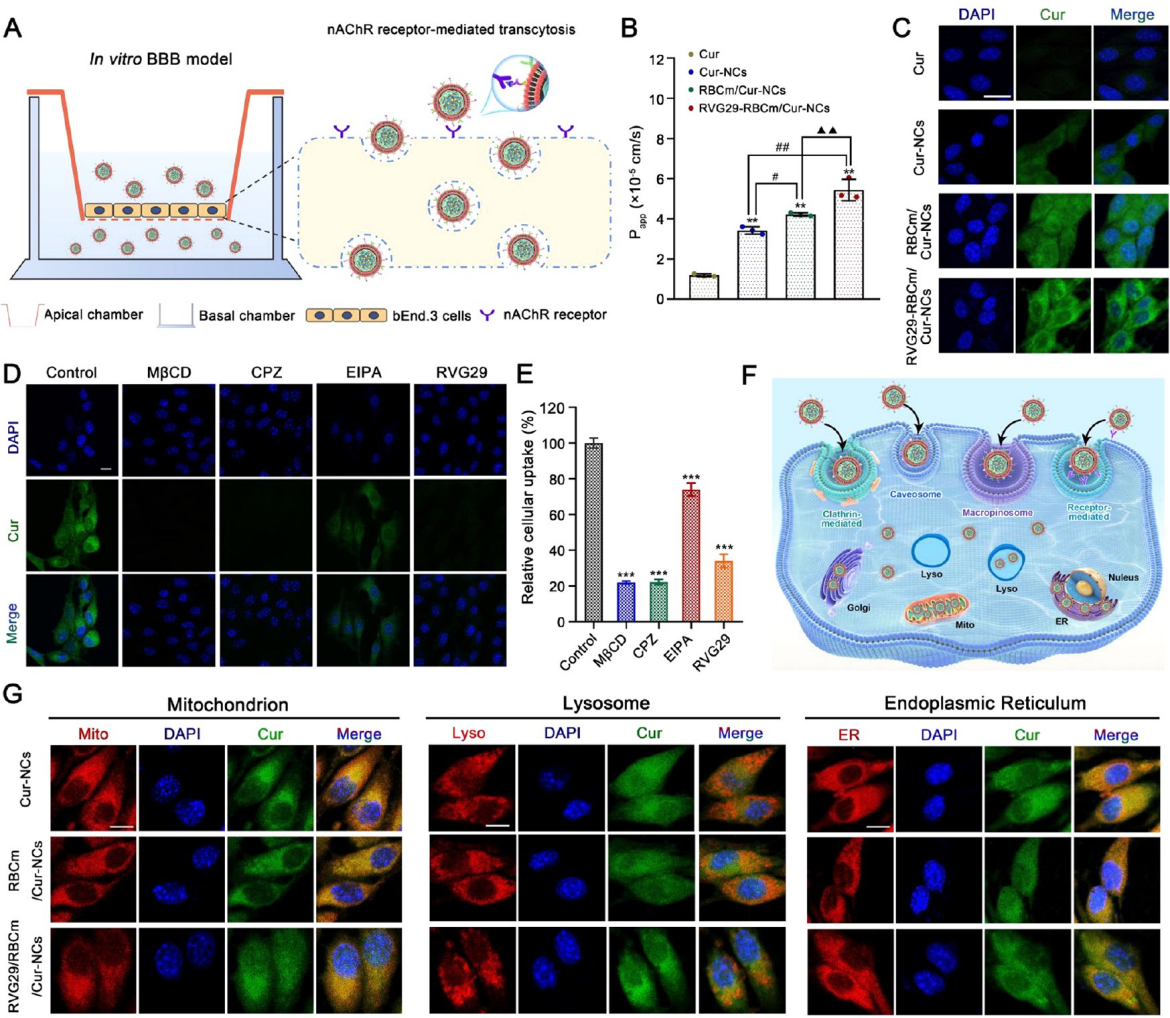
Cellular uptake and transportation of Cur formulations in bEnd.3 cells. (A) In vitro BBB model established using bEnd.3 cells. (B) Apparent permeability coefficient (*n* = 3). Cur was diluted in dimethyl sulfoxide (DMSO) as the control. *P < 0.05 and **P < 0.01 vs the Cur group. ^#^*P* < 0.05 and ^##^*P* < 0.01 vs the Cur-NCs group. ^▲^*P* < 0.05 and ^▲▲^*P* < 0.01 vs the RBCm/Cur-NCs group. (C) Uptake of various formulations by bEnd.3 cells after incubation with Cur concentrations of 100 μM for 3 h. Scale bar: 20 μm. (D) Representative CLSM images obtained after treatment with different inhibitors. (E) Fluorescence quantification after treatment with different inhibitors. Scale bar: 10 μm. ***P < 0.001 vs the control group. (F) Cellular uptake and transport of RVG29-RBCm/Cur-NCs in bEnd.3 cells. (G) Colocalization of RVG29-RBCm/Cur-NCs with the mitochondria, lysosomes, and endoplasmic reticulum. Scale bar: 10 μm.

The Transwell assay was conducted to evaluate the BBB permeability of different Cur formulations as well as the potential targeting ability of RVG29-RBCm/Cur-NCs (Figure 3A). No significant cytotoxicity was observed after the incubation of Cur formulations with bEnd.3 cells (Figure S11). Evaluation of the apparent permeability coefficient (*P*_app_) showed that Cur nanopreparations significantly enhanced the transport capacity of Cur across a monolayer of bEnd.3 cell. This was found to improve Cur solubility achieved through the nanopreparation (Figure 3B). Compared with the Cur-NCs group, the RBCm/Cur-NCs group showed significantly higher Cur transport, likely owing to increased biocompatibility and beneficial interactions with outer phospholipids. Moreover, RVG29-RBCm/Cur-NCs showed the best ability to traverse the in vitro BBB. It is speculated that RVG29 targets the nAChR receptors expressed on the surface of bEnd.3 cells to induce receptor-mediated transcellular transport. Moreover, there was no significant change in the transepithelial electrical resistance (TEER) value in each group before and after transport (Figure S12), indicating that the monolayer of bEnd.3 cells remained intact and transportation did not occur via the disruption of the BBB.

### Cellular Uptake of RVG29-RBCm/Cur-NCs in bEnd.3 Cells

Cellular uptake was examined in bEnd.3 cells after incubation with different concentrations of Cur preparations for different durations. The experiments showed that uptake was low after incubation with Cur (physical mixture of Cur and PVP) (Figure 3C and Figure S13). However, Cur-NCs improved Cur uptake in a concentration- and time-dependent manner. As the incubation period increased, the fluorescence signal in bEnd.3 cells incubated with RBCm/Cur-NCs became stronger than that in cells incubated with Cur-NCs. At all concentrations and incubation periods, the RVG29-RBCm/Cur-NCs group exhibited the strongest fluorescence, indicating that modification with the targeting peptide RVG29 could promote the internalization of Cur. These results illustrated that the increase in Cur transport across the BBB in vitro is due to the increased cellular uptake of Cur, which promotes Cur transcytosis.

Endocytosis inhibitors were used to study the mechanism underlying RVG29-RBCm/Cur-NCs uptake by bEnd.3 cells (Table S1). Untreated cells incubated with RVG29-RBCm/Cur-NCs were designated as the control group, and their fluorescence intensity was considered 100%. Subsequently, the percentage of Cur fluorescence intensity under different inhibition conditions was measured. All inhibitors significantly reduced Cur uptake in bEnd.3 cells (Figure 3D and 3E). The uptake observed after treatment with MβCD, which inhibits caveolin-mediated endocytosis, was only 21.00%. When cells were treated with CPZ, which inhibits clathrin-mediated endocytosis, the uptake rate decreased to 22.22%. Treatment with EIPA, a macropinocytosis inhibitor, also affected the endocytosis of RVG29-RBCm/Cur-NCs, reducing uptake to 74.04%. Notably, preincubation with an excess of RVG29 led to the saturation of nAChR receptors on the surface of bEnd.3 cells, leading to competitive inhibition with RVG29-RBCm/Cur-NCs and reducing Cur fluorescence significantly to 34.01%.

These results suggested that RVG29-RBCm/Cur-NC uptake by bEnd.3 cells involves multiple endocytosis mechanisms, including receptor-mediated uptake. The results also confirmed that RVG29-modified RBCm/Cur-NCs could bind to the nAChR receptors on the surface of bEnd.3 cells, thus enhancing the intracellular concentration of Cur and transcytosis.

### Intracellular Trafficking of Cur Formulations

In order to explore the intracellular fate of Cur nanopreparations after uptake by bEnd.3 cells, their colocalization with subcellular organelles was examined using CLSM. Cur-NCs, RBCm/Cur-NCs, and RVG29-RBCm/Cur-NCs all showed a large degree of fluorescence overlap with mitochondria, suggesting that these nanoparticles were transported to mitochondria (Figure 3G). The pathogenesis of PD is closely related to mitochondrial dysfunction.^25^ Thus, owing to this large colocalization, the effect of the drugs on cellular mitochondria may be enhanced. Nanodrug delivery systems mainly enter cells through lysosome-related endocytosis.^26^ In this study, lysosomal colocalization experiments showed that the RVG29-RBCm/Cur-NCs group had the lowest colocalization with lysosomes (Figure 3G). The endoplasmic reticulum plays an important role in endocytosis, especially in intercellular transport.^26^ The fluorescence signals of Cur-NCs, RBCm/Cur-NCs, and RVG29-RBCm/Cur-NCs overlapped with the endoplasmic reticulum (Figure 3G). This suggested that in bEnd.3 cells, the transport of the Cur nanopreparations occurred via the endocytosis pathway, consistent with the results of the uptake mechanism experiment.

In summary, the high uptake of RVG29-RBCm/Cur-NCs by bEnd.3 cells appeared to be related to targeted modification using RVG29. Caveolin- and clathrin-mediated endocytosis and macropinocytosis also appeared to be involved in drug uptake. Finally, nanoparticles were mainly transported to the mitochondria and endoplasmic reticulum, and transport to lysosomes was less frequent (Figure 3F).

### In Vitro Neuroprotective Effect of RVG29-RBCm/Cur-NCs

**Figure 4:**
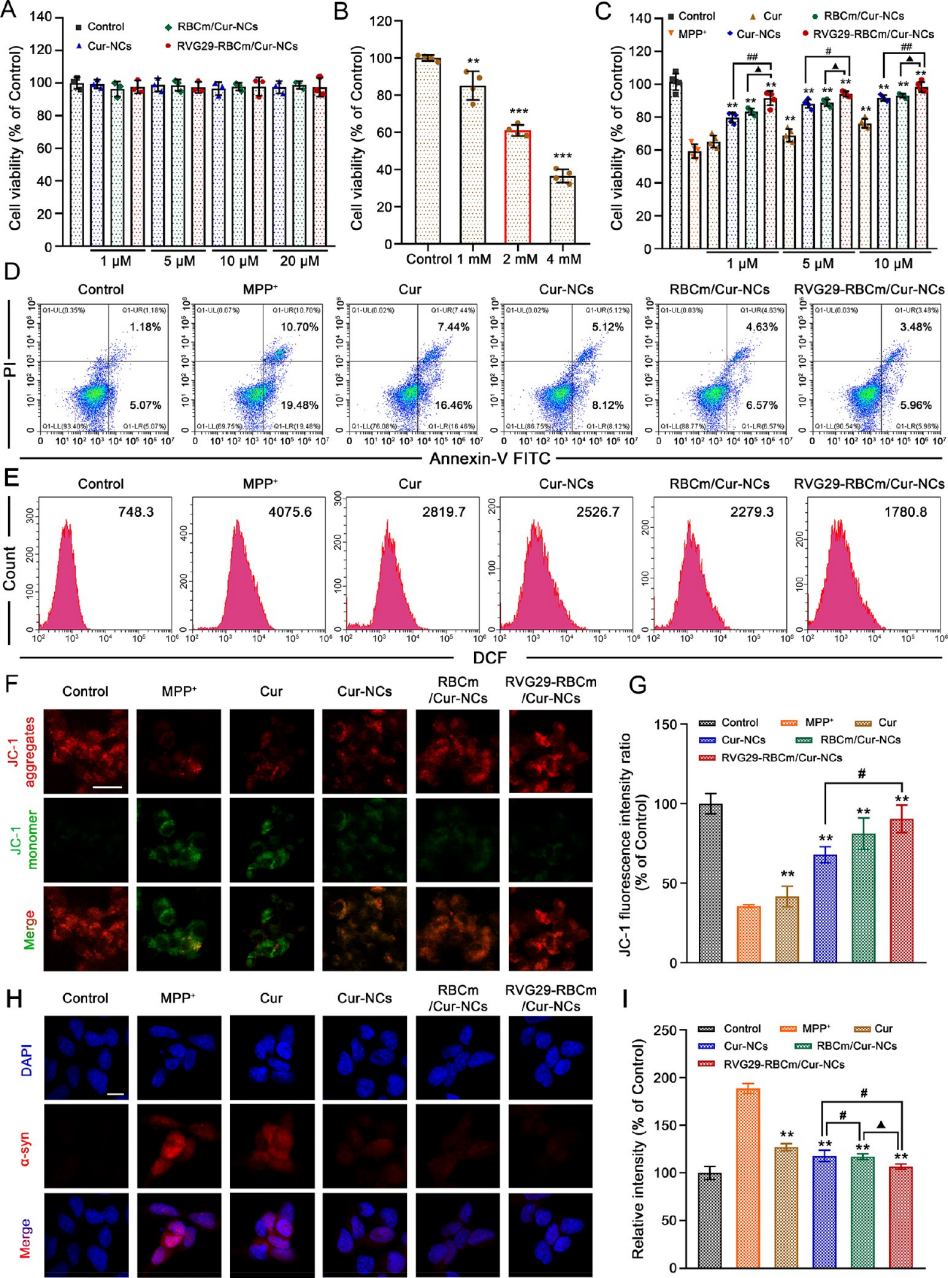
Neuroprotective effect of RVG29-RBCm/Cur-NCs on SH-SY5Y cells damaged after MPP^+^ treatment. (A) Cytotoxicity evaluation of Cur formulations in SH-SY5Y cells (*n* = 3). (B) Neurotoxicity of different concentrations of MPP^+^ in SH-SY5Y cells (*n* = 3). **P < 0.01 and ***P < 0.001 with respect to the control group. (C) Cell viability after treatment with various Cur formulations (*n* = 5). *P < 0.05 and **P < 0.01 with respect to the MPP^+^ group. ^#^*P* < 0.05 and ^##^*P* < 0.01 with respect to the Cur-NCs group. ^▲^*P* < 0.05 and ^▲▲^*P* < 0.01 with respect to the RBCm/Cur-NCs group. (D) Apoptosis assay using flow cytometry. (E) ROS production in SH-SY5Y cells detected using the DCFH-DA assay. (F) Mitochondrial membrane potential of SH-SY5Y cells before and after incubation with MPP^+^ and pretreatment with RVG29-RBCm/Cur-NCs. Scale bar: 25 μm. (G) JC-1 fluorescence intensity ratios. (H) Immunofluorescence images of α-syn expression after pretreatment with RVG29-RBCm/Cur-NCs in vitro PD model. Scale bar: 10 μm. (I) Relative fluorescence intensity of α-syn.

The Cur formulations showed good biocompatibility in SH-SY5Y cells at Cur concentrations of 1–20 μM (Figure 4A). Next, the effect of different concentrations of 1-methyl-4-phenylpyridinium ion (MPP^+^) on the viability of SH-SY5Y cells was explored to identify the appropriate modeling concentration for subsequent evaluations. The final optimized MPP^+^ concentration was 2 mM. At this concentration, enough cytotoxicity (inhibition of cell viability = ∼40%) could be achieved without large amounts of cell damage that would affect subsequent examinations (Figure 4B). When the cells were pretreated with different Cur formulations, Cur-NCs, RBCm/Cur-NCs, and RVG29-RBCm/Cur-NCs significantly improved the viability of SH-SY5Y cells treated with 2 mM MPP^+^ (Figure 4C). Notably, at the same therapeutic concentration (1, 5, and 10 μM), treatment with RVG29-RBCm/Cur-NCs could significantly increase the survival rate (91.60%, 94.08%, and 98.26%, respectively). The results of live/dead cell staining also verified the higher rates of cell survival after treatment with Cur nanopreparations (Figure S14).

Next, the antiapoptosis effect of RVG29-RBCm/Cur-NCs was examined using Annexin V-FITC/PI staining. The apoptosis percentage was calculated based on early and late apoptosis rates.^27^ After MPP^+^ treatment, the apoptosis percentage was as high as 30.18% (Figure 4D). After treatment with RVG29-RBCm/Cur-NCs, the apoptosis rate was reduced significantly to 9.44%. This reduction was greater than that observed with Cur (23.90%), Cur-NCs (13.24%), and RBCm/Cur-NCs (11.20%), demonstrating the enhanced neuroprotection offered by RVG29-RBCm/Cur-NCs. MPP^+^ accumulation disturbs the mitochondria, resulting in the production of excess cytotoxic ROS, which is the primary cause of neuronal apoptosis.^28^ The intracellular ROS levels were lower in the Cur treatment groups than those in the MPP^+^ group, and the therapeutic effect was the highest for RVG29-RBCm/Cur-NCs (Figure 4E).

Finally, the depolarization of the mitochondrial membrane in SH-SY5Y cells was studied using JC-1 staining.^29^ In normal SH-SY5Y cells, a strong red fluorescence was observed, indicating a high mitochondrial membrane potential. In contrast, MPP^+^-treated cells showed a green fluorescence signal, indicating a reduced mitochondrial membrane potential—a sign of early apoptosis (Figure 4F). Pretreatment with RVG29-RBCm/Cur-NCs could prevent the depolarization of the mitochondrial membrane, increasing the JC-1 fluorescence ratio to 90.45% (Figure 4G). This restoration was consistent with the antiapoptotic effect observed in the flow cytometric analysis. Furthermore, RVG29-RBCm/Cur-NCs also effectively inhibited the high abnormal expression of α-syn induced by MPP^+^ treatment (Figure 4H and 4I).

These results showed that the Cur nanopreparations could enhance the neuroprotective effect of Cur. Cell survival was better in the RVG29-RBCm/Cur-NCs group than that in the RBCm/Cur-NCs and Cur-NCs groups. This could be related to modification with RVG29. The RVG29-RBCm/Cur-NCs targeted the nAChR receptors expressed on SH-SY5Y cells and entered cells through the receptor-mediated pathway, subsequently playing a neuroprotective role.

### In Vivo Safety Investigation

**Figure 5:**
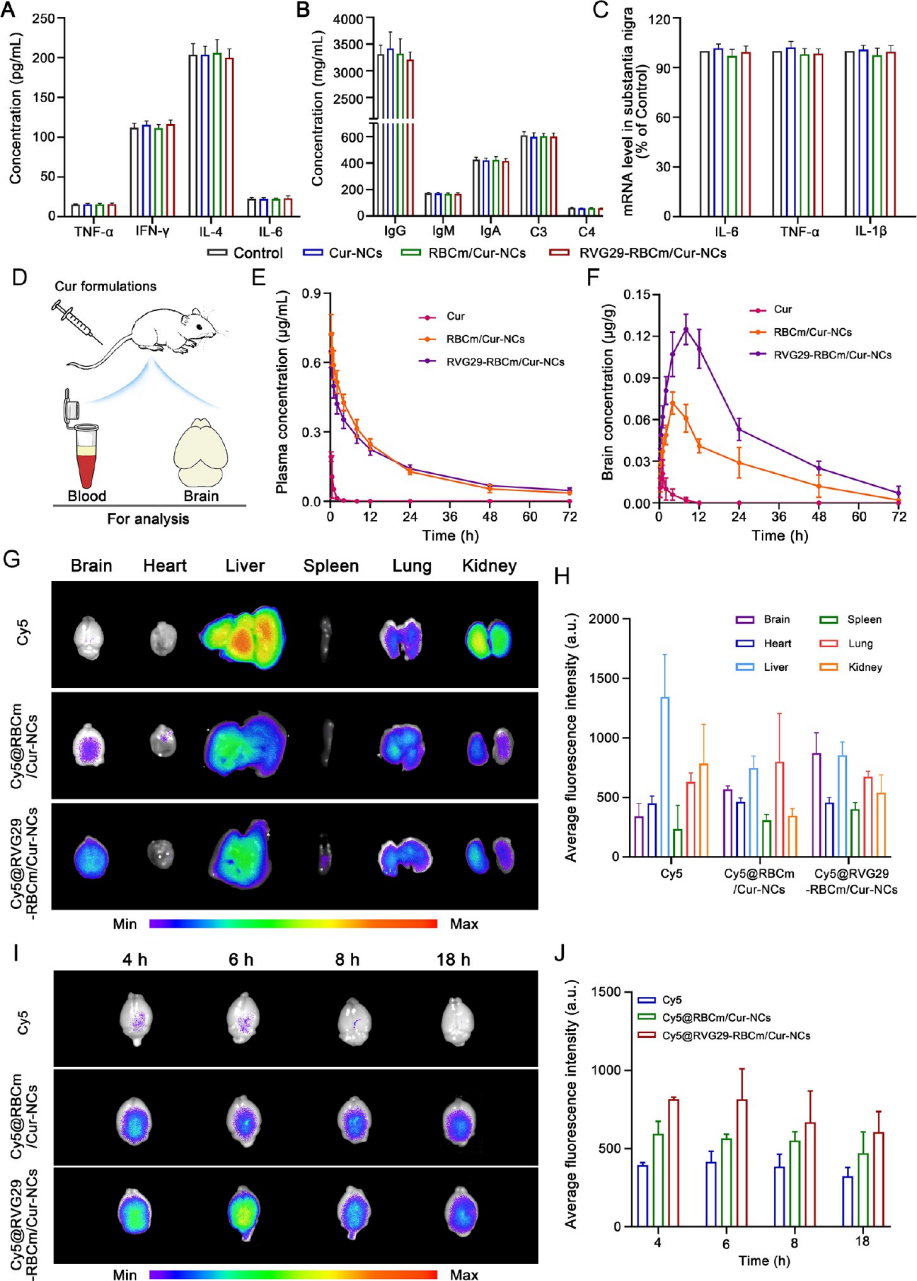
In vivo safety assessment and distribution of RVG29-RBCm/Cur-NCs. (A and B) Evaluation of immunogenicity. Serum levels of (A) cytokines and (B) immune antibodies and complements (*n* = 4). (C) Levels of inflammatory factors in the midbrain. (D–F) Pharmacokinetic evaluation (*n* = 4). (D) Blood and brain samples were collected and analyzed to understand drug pharmacokinetics in the (E) plasma and (F) brain. (G–J) In vivo distribution (*n* = 3). (G) Representative distribution of Cy5 in major organs at 2 h. (H) Quantification of fluorescence intensity in the major organs. (I) Representative fluorescence images of brain tissue at different time points. (J) Quantification of fluorescence intensity in the brain. *P < 0.05 and **P < 0.01 with respect to the Cy5 group. ^#^*P* < 0.05 and ^##^*P* < 0.01 with respect to the RVG29-RBCm/Cur-NCs group.

Before intravenous administration in vivo, a hemolysis study was performed in vitro using the prepared Cur nanoformulations. As shown in Figure S15, Cur-NCs, RBCm/Cur-NCs, and RVG29-RBCm/Cur-NCs did not cause significant hemolysis (<5%), and they thus appeared suitable for intravenous administration. Next, serum obtained from healthy mice after the 15-day administration period was examined. The levels of TNF-α and IFN-γ, which are Th1 cytokines and are associated with the cellular immune response, were examined. The levels of Th2 cytokines (including IL-4 and IL-6), which mediate the immune response,^30^ were also examined. Fortunately, no abnormal changes in the levels of TNF-α, IFN-γ, IL-4, and IL-6 were observed after treatment with the Cur nanopreparations (Figure 5A). Similarly, there was no significant change in the levels of IgG, IgM, IgA, and complement C3 and C4 in all groups (Figure 5B). These results indicated that the RBCm does not cause a significant immunogenic response in vivo. The surface antigens present on the RBCm do not cause immune rejection. Instead, they provide a double-layer lipid barrier for nanoparticles, and the CD47 expressed on the RBCm may even help in escape from monitoring by immune cells.

During brain-targeted drug delivery, the potential inflammatory response in the brain is also a concern. Experiments showed that the mRNA levels of inflammatory factors (TNF-α, IL-1β, and IL-6) in the midbrain and striatum were similar between the treatment and the control groups (Figure 5C and Figure S16). These results showed that the prepared Cur-NCs, RBCm/Cur-NCs, and RVG29-RBCm/Cur-NCs had good biosafety in vivo.

### In Vivo Pharmacokinetic Study and Biodistribution of RVG29-RBCm/Cur-NCs

A pharmacokinetic study was performed by collecting plasma and brain tissue in order to explore the in vivo distribution of different Cur formulations (Figure 5D). Regarding plasma pharmacokinetic parameters (Figure 5E and Table S2), the blood circulation half-life of RVG29-RBCm/Cur-NCs (20.036 ± 1.573 h) was significantly longer than that of free Cur (0.448 ± 0.036 h) and RBCm/Cur-NCs (15.984 ± 1.285 h). This suggested that RVG29-RBCm/Cur-NCs had a longer blood circulation life. Notably, both RBCm/Cur-NCs and RVG29-RBCm/Cur-NCs exhibited a longer half-life than that of the reported Cur nanoparticles (*T*_1/2_ of 6.22 h),^31^ which further confirmed the superiority of RBCm coating. Moreover, higher peak Cur levels were achieved with RVG29-RBCm/Cur-NCs than with free Cur (0.596 ± 0.045 vs 0.106 ± 0.087 μg/mL). The area under the curve for Cur levels in the plasma from time zero to *t* (*AUC*_0–*t*_) (10.186 ± 0.904 vs 0.177 ± 0.013 μg·h/mL) and mean residence time (21.252 ± 1.892 vs 0.864 ± 0.074 h) were also higher. These results demonstrate that RVG29-RBCm/Cur-NCs show lower capture by mononuclear phagocytes and act as a long-circulating reservoir for drug delivery.

In the brain (Figure 5F and Table S2), RVG29-RBCm/Cur-NCs showed better in vivo circulation and retention behavior than did Cur and RBCm/Cur-NCs. Compared with RBCm/Cur-NCs, RVG29-RBCm/Cur-NCs had a longer blood circulation half-life (12.849 ± 1.115 vs 15.236 ± 1.340 h; 1.19-fold). Further, they required a longer time to reach peak Cur levels (4.500 ± 1.000 vs 7.500 ± 1.000 h; 1.67-fold), showed higher peak Cur levels (0.072 ± 0.009 vs 0.125 ± 0.015 μg/g; 1.74-fold), and had higher *AUC*_0–*t*_ values (1.744 ± 0.151 vs 3.550 ± 0.332 μg.h/g; 2.04-fold) and mean residence durations (20.076 ± 1.896 vs 21.841 ± 1.777 h; 1.09-fold). These results confirmed that due to RVG29 modification, RVG29-RBCm/Cur-NCs not only circulate for longer in vivo but also allow targeted drug delivery from the blood to the brain.

To verify the brain-targeting ability of RVG29-RBCm/Cur-NCs in vivo, cell membranes were labeled with Cy5 and the accumulation of the biomimetic nanoparticles in the brain was evaluated. Compared with free Cy5, biomimetic nanoparticles showed higher accumulation in the brain, likely due to the long circulation time and immune escape characteristics provided by the RBCm. Importantly, given the target ligand modification, Cy5@RVG29-RBCm/Cur-NCs exhibited an ideal distribution in vivo. These biomimetic nanodecoys were primarily targeted to the brain with little accumulation in the liver and kidneys (Figure 5G and 5H), while the free Cy5 was quickly taken up by the liver and kidney. Further, Cy5@RVG29-RBCm/Cur-NCs showed the strongest fluorescence in the brain at each time point with considerable fluorescence even after 18 h (Figure 5I and 5J). This suggested that coating NCs with the RBCm prolonged their circulation. The RVG29 peptide promoted the localization of the biomimetic NCs in the brain and enhanced their accumulation, consistent with pharmacokinetic findings.

### Recovery of Behavioral Defects in PD Mice

Mice were treated with continuous 1-methyl-4-phenyl-1,2,3,6-tetrahydropyridine (MPTP) administration to induce PD-like symptoms (e.g., bradykinesia, rigidity, rest tremor, and postural instability).^32^ MPTP administration is a classic approach for generating animal models of PD, and it simulates the process of α-syn aggregation and DA neuron degeneration.^33^ After passing through the BBB, MPTP is oxidized to MPP^+^, which enters DA neurons through the DA transporter. MPP^+^ accumulates in the mitochondria of DA neurons and inhibits the complex I electron transport chain, causing a series of metabolic abnormalities.^34^

**Figure 6:**
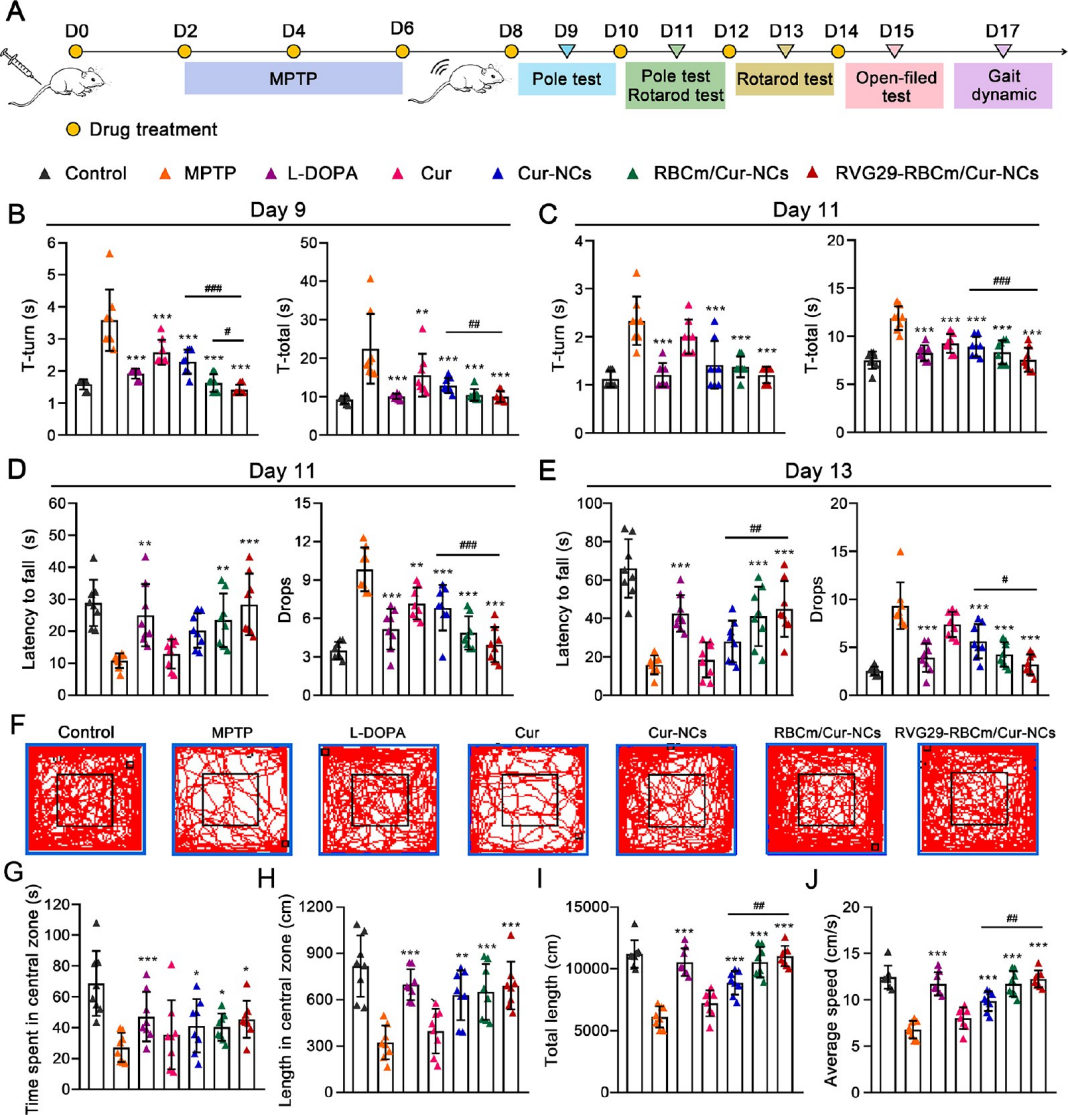
Behavioral analysis (*n* = 8). (A) Timeline for the establishment of the MPTP-induced PD mouse model and the administration of Cur formulations. (B and C) Pole test on (B) day 9 and (C) day 11. (D and E) Rotarod test on (D) day 11 and (E) day 13. (F–J) Open-field test. (F) Respective movement paths. (G) Time spent in the central zone. (H) Length traveled in the central zone. (I) Total length. (J) Average speed. *P < 0.05, **P < 0.01, and ***P < 0.001 with respect to the MPTP group. ^#^*P* < 0.05 and ^##^*P* < 0.01 with respect to the RVG29-RBCm/Cur-NCs group.

During the pharmacodynamic study (Figure 6A), all treated mice underwent PD-related behavioral tests. In the pole test, the therapeutic treatment groups showed significantly lower T-turn and T-total values on day 9 and day 11 than did the MPTP group. This indicated that the treatments attenuated motor retardation symptoms in PD mice (Figure 6B and 6C). Coordination and balance were assessed in PD mice using the rotarod test. The mice receiving therapeutic treatment remained on the rotating rod for a longer period and showed fewer drops on day 11 and day 13 (Figure 6D and 6E). An open-field test was conducted on day 15 to evaluate spontaneous movement in the PD mice. The representative movement paths suggested that MPTP-treated mice traveled the shortest distance (Figure 6F). Further analysis showed that the movement speed was lowest in the MPTP group, and this group also showed the shortest movement path and time spent in the central area. The therapeutic effect of Cur was weak, whereas treatment with Cur-NCs, RBCm/Cur-NCs, and RVG29-RBCm/Cur-NCs prolonged the time spent by mice in the central zone and significantly improved the total distance traveled and average speed (Figure 6G–J).

**Figure 7:**
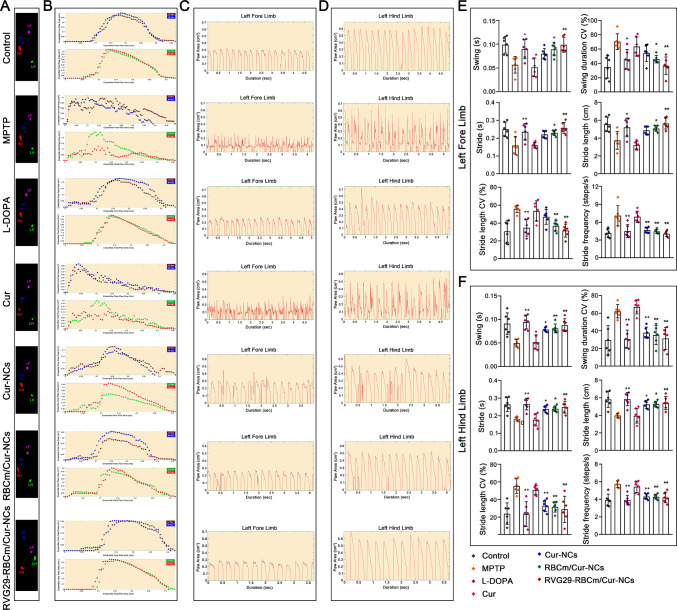
Gait dynamics analysis (*n* = 6). (A and B) (A) Representative images of posture and (B) paw area–time curves. (C and D) Representative gait signal from the (C) left forelimbs and (D) hindlimbs of mice after different treatment. (E and F) Gait parameters including swing, swing duration CV, stride, stride length, stride length CV, and stride frequency of the (E) left forelimbs and (F) hindlimbs of the mice in C and D. *P < 0.05 and **P < 0.01 with respect to the MPTP group.

Gait asymmetry is a feature that causes disability and injury during PD progression. Not surprisingly, MPTP-treated PD mice developed gait abnormalities: they showed a significant decrease in limb stride length and took more steps in a shorter period of time (Figure 7, Figure S17, and Figure S18). After treatment with the different Cur nanopreparations, the gait parameters of mice (Table S3) showed an obvious improvement. The swing, coefficient of variation in swing duration, stride, stride length, coefficient of variation in stride length, and stride frequency all increased after treatment. These results showed that Cur nanopreparations can attenuate the behavioral defects observed in PD mice. Notably, RVG29-RBCm/Cur-NCs showed the greatest effect in attenuating behavioral defects. This suggested that treatment with RVG29-RBCm/Cur-NCs allowed effective reversal of behavioral deficiencies in PD mice.

Weight-monitoring experiments showed that mice experienced slight weight loss during the MPTP treatment period. Subsequently, their weight increased normally during drug administration, and no signs of toxicity were observed based on their eating behavior and activities (Figure S19).

### Alleviation of Neuronal Damage in PD Mice

**Figure 8:**
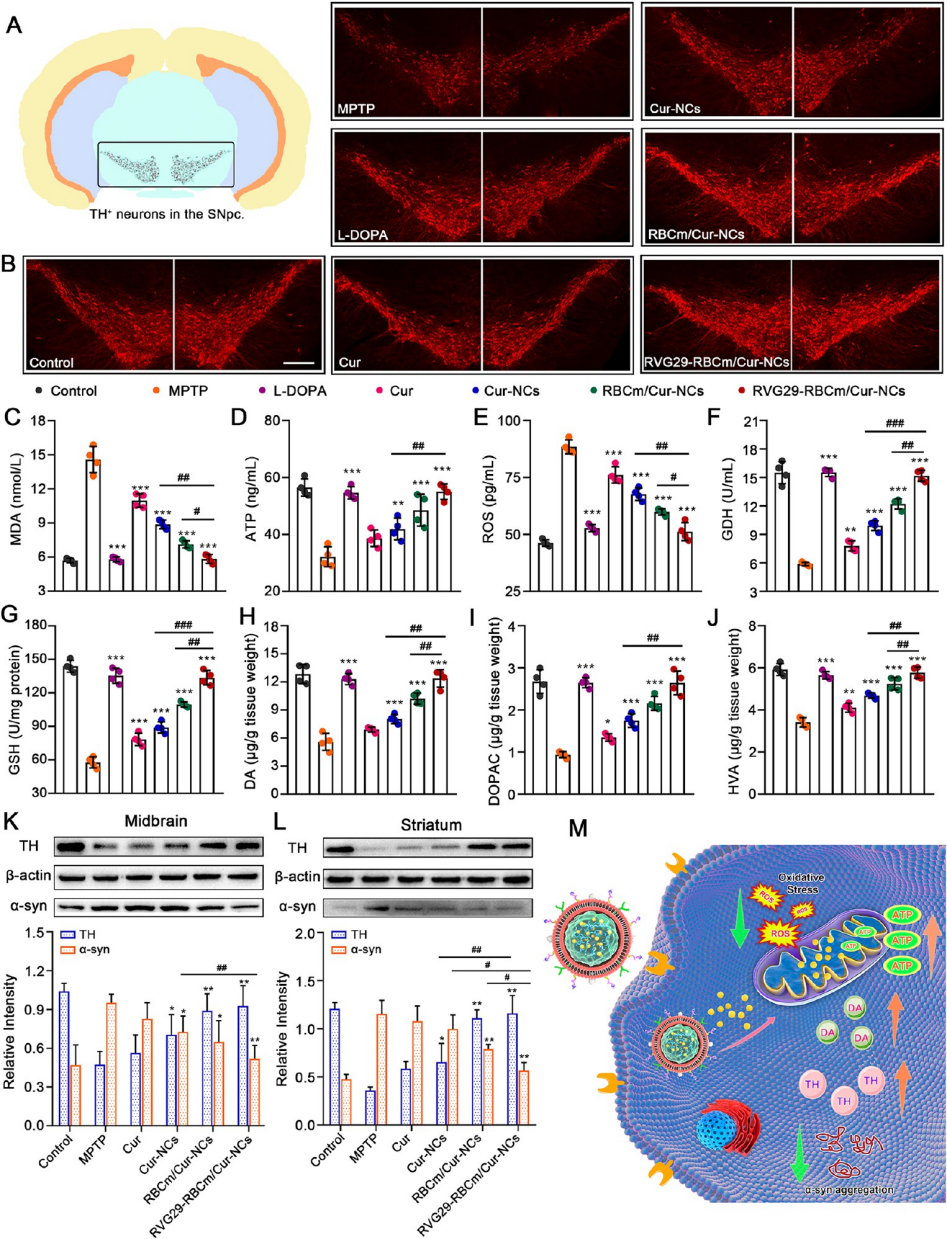
Neuroprotective effect of RVG29-RBCm/Cur-NCs in vivo. (A) Schematic diagrams showing the location of TH^+^ neurons in the SNpc. (B) Representative images of TH^+^ immunofluorescence staining in brain sections. Scale bar: 250 μm. (C–G) Oxidative stress and mitochondrial function (*n* = 4). Levels of (C) MDA, (D) ATP, (E) ROS, and (F) GDH in the midbrain and levels of (G) GSH in the striatum were measured. *P < 0.05, **P < 0.01 and ***P < 0.001 with respect to the MPTP group. ^#^*P* < 0.05 and ^##^*P* < 0.01 with respect to the RVG29-RBCm/Cur-NCs group. MDA, malondialdehyde; ATP, adenosine triphosphate; GDH, glutamate dehydrogenase; GSH, glutathione. (H–J) Dopamine metabolism in the striatum, including the (H) dopamine level, (I) DOPAC level, and (J) HVA level (*n* = 4). *P < 0.05 and **P < 0.01 with respect to the MPTP group. ^#^*P* < 0.05 and ^##^*P* < 0.01 with respect to the RVG29-RBCm/Cur-NCs group. (K and L) Expression levels of TH and α-syn in the (K) midbrain and (L) striatum (*n* = 3). *P < 0.05 and **P < 0.01 with respect to the MPTP group. ^#^*P* < 0.05 and ^##^*P* < 0.01 with respect to the RVG29-RBCm/Cur-NCs group.

MPTP-induced neurotoxicity promotes the loss of TH^+^ neurons, and the degree of loss reflects the reduction in DA levels.^35^ Therefore, immunofluorescence staining was performed to detect the number of TH^+^ neurons in the SNpc in mice from various treatment groups (Figure 8A). Compared with the control group, the MPTP group showed a significantly diminished population of TH^+^ neurons (∼65%) (Figure 8B and Figure S20). This was due to MPTP-induced injury to DA neurons. The quantification of TH^+^ neurons showed that treatment with Cur-NCs, RBCm/Cur-NCs, and RVG29-RBCm/Cur-NCs could increase the number of TH^+^ neurons; after treatment with these preparations, the number of TH^+^ neurons was restored to 84.18%, 89.64%, and 97.38%, respectively, of that in the control group (Figure 8B and Figure S20).

MPTP intervention promoted oxidative stress, increasing MDA levels and decreasing superoxide dismutase (SOD)/glutathione (GSH) activity.^36^ Compared with the MPTP group, the RVG29-RBCm/Cur-NCs group showed significantly reduced MDA levels, both in the midbrain and in the striatum (Figure 8C and Figure S21A). Moreover, in the RVG29-RBCm/Cur-NCs group, the SOD and GSH activity in the striatum increased to 97.57% and 92.84% of that in the control group, respectively (Figure S21B and Figure 8G). MPP^+^ accumulation occurred in DA neurons, impairing mitochondria. This was followed by excessive ROS production, which could interfere with or even destroy DA neurons.^37^ The ATP level in the MPTP group was only 56.97% of that in the control group. However, the ATP level in the RVG29-RBCm/Cur-NCs group was as high as 97.43% (Figure 8D). After MPTP treatment, ROS production in the mouse midbrain was about 1.9 times higher than that in untreated mice (Figure 8E). However, RVG29-RBCm/Cur-NCs treatment decreased ROS levels to 57.89% of that in the MPTP group.

Glutamate dehydrogenase (GDH) is an enzyme in the mitochondrial matrix and is also a marker of mitochondrial membrane integrity.^38^ As expected, RVG29-RBCm/Cur-NCs reversed the MPTP-induced decline in GDH (Figure 8F). These results suggested that RVG29-RBCm/Cur-NCs can reduce MPTP-induced mitochondrial dysfunction.

PD-like symptoms are accompanied by reduced DA metabolism. The levels of dopamine and its metabolites 3,4-dihydroxyphenylacetic acid (DOPAC) and homovanillic acid (HVA) in the striatum of PD mice were only 43.55%, 35.09%, and 57.70% of those in control mice, respectively (Figure 8H–J). After therapeutic treatment, these levels increased to a certain degree. Among the Cur nanopreparation groups, RVG29-RBCm/Cur-NCs provided the highest increase in dopamine and its metabolites, restoring the levels of dopamine, DOPAC, and HVA to 96.42%, 98.80%, and 97.48%, respectively (Figure 8H–J). These results confirmed that RVG29-RBCm/Cur-NCs can reduce the MPTP-induced damage to the DA system.

In order to study pathological changes in the brain after the induction of PD models, the expression levels of TH and α-syn were detected using Western blot. Relative to the MPTP group, the RVG29-RBCm/Cur-NCs group showed significantly increased levels of TH, both in the midbrain and in the striatum (Figure 8K and 8L). These findings were consistent with those of TH^+^ immunofluorescence. Furthermore, RVG29-RBCm/Cur-NCs significantly attenuated the abnormal elevations in α-syn aggregation. Hence, it appeared that the anti-Parkinsonian efficacy of RVG29-RBCm/Cur-NCs was also mediated by effects on the pathogenesis of PD, including TH deficiency and α-syn aggregation. Together, the therapeutic effect of RVG29-RBCm/Cur-NCs could be attributed to the enhanced solubility and long circulation half-life of Cur, BBB-targeting modifications that allow efficient transport, and the inhibition of mitochondrial dysfunction and abnormal protein expression.

### In Vivo Biocompatibility

After the 15-day treatment period, mice were sacrificed and their blood and major organs were collected for further analysis. Blood routine and biochemistry parameters were similar between the control and the treatment groups (*P* > 0.05) (Figures S22 and S23). In addition, no abnormalities were observed in the organ indexes of the heart, liver, spleen, lung, and kidney after treatment (Figure S24). Furthermore, hematoxylin and eosin (H&E) staining also indicated the absence of any organ damage (Figure S25). These results indicated that the Cur formulations did not cause any inflammation, infection, or organ injury and were therefore biocompatible. On the basis of the findings of these brain-targeted biomimetic nanodecoys, RVG29-RBCm/Cur-NCs can not only have good biocompatibility but also significantly prolong circulation time in vivo and directly deliver Cur into the brain, which help to reduce administration times and improve the compliance of patients.

## Conclusions

In this study, brain-targeted biomimetic nanodecoys, RVG29-RBCm/Cur-NCs, were developed for PD therapy. The goal of this system was to prolong the blood retention, overcome the BBB, and improve the bioavailability of potential anti-PD drugs. RVG29-RBCm/Cur-NCs penetrated the BBB and accumulated on neurons by binding to nAChR receptors. They exhibited satisfactory neuroprotective effects in an MPTP/MPP^+^-induced mouse model of PD, providing not only improvements in motor behavior in PD mice but also attenuating the pathological decrease in TH^+^ neurons, inhibiting abnormal α-syn aggregation, enhancing DA levels, and reversing mitochondrial dysfunction in the brain. Moreover, the RVG29-RBCm/Cur-NCs showed excellent biocompatibility. Taken together, the findings of this study demonstrate that RVG29-RBCm/Cur-NCs represent a promising strategy for brain-targeted drug delivery in the treatment of PD and other neurodegenerative diseases.

## Supporting Information Available

Characterization of Cur-NCs; ^1^H NMR spectra; serum stability; TEM images; UV–vis absorption spectra; cytotoxicity evaluation; images of cellular uptake; TEER values; live/dead staining; hemolysis study; levels of inflammatory factors; gait parameters; body weight; number of TH^+^ neurons; MDA and SOD levels; blood routine analysis; biochemical analysis of blood; organ index; H&E images, and pharmacokinetic parameters (PDF)
