# Supplementary material for: Brain-Targeted Biomimetic Nanodecoys with Neuroprotective Effects for Precise Therapy of Parkinson’s Disease
Source: ACS Cent Sci. 2022 Sep 13;8(9):1336–49. doi: 10.1021/acscentsci.2c00741 (PMC9523773; doi:10.1021/acscentsci.2c00741)
Supplement: Supplementary file 1 — oc2c00741_si_001.pdf [file oc2c00741_si_001.pdf]

Supporting Information

Brain-Targeted Biomimetic Nanodecoys with Neuroprotective Effects for Precise Therapy of Parkinson's Disease

Yao Liu[1], Jingshan Luo[1], Yujing Liu[1], Wen Liu[2], Guangtao Yu[3], Yuting Huang[4], Yu Yang[4]*, Xiaojia Chen[2]*, Tongkai Chen[1]*

[1]*Science and Technology Innovation Center, Guangzhou University of Chinese Medicine, Guangzhou 510405, China*

[2]*State Key Laboratory of Quality Research in Chinese Medicine, Institute of Chinese Medical Sciences, University of Macau, Macau 999078, China*

[3]*Stomatological Hospital, Southern Medical University, Guangzhou 510280, China.*

[4]*Institute of Molecular Medicine (IMM), Renji Hospital, School of Medicine, Shanghai Jiao Tong University, Shanghai 200240, China.*

* To whom correspondence should be addressed:

1. Tongkai Chen, Ph.D.

Science and Technology Innovation Center, Guangzhou University of Chinese Medicine, 12 Jichang Road, Guangzhou 510405, China

Tel.: +86 20 36585707

E-mail: chentongkai@gzucm.edu.cn

2. Xiaojia Chen, Ph.D.

Institute of Chinese Medical Sciences, University of Macau, Room 6007, N22, Taipa, Macau SAR

Tel.: (853) 88224915

E-mail: xiaojiachen@um.edu.mo

3. Yu Yang, Ph.D.

Institute of Molecular Medicine (IMM), Renji Hospital, School of Medicine, Shanghai Jiao Tong University, Shanghai 200240, China.

E-mail: yuyang@shsmu.edu.cn

Experimental section

Materials

Curcumin (Cur; purity ≥ 98%) was procured from Nantong Feiyu Biological Technology Co., Ltd. (Jiangsu, China). Acetone was purchased from Guangzhou Chemical Reagent Factory (Guangzhou, China). PVP K30 was bought from Ashland. Levodopa (L-DOPA), 1-methyl-4-phenylpyridinium ion (MPP^+), and the rabbit anti-TH antibody were obtained from Sigma-Aldrich (St. Louis, USA). 1-methyl-4-phenyl-1,2,3,6-tetrahydropyridine hydrochloride (MPTP·HCl) was purchased from MedChemExpress (New Jersey, USA). Rabbit anti-α-syn antibodies were purchased from Abcam (Cambridge, UK). DMEM, DMEM/F12, and fetal bovine serum (FBS) were obtained from Gibco (USA). All the chemicals were of analytical grade and were used without further treatment. Polycarbonate porous membrane syringe filters were provided by Whatman (USA).

Cell culture

A mouse brain microvascular endothelial cell line (bEnd.3) and mouse monocyte macrophages (RAW264.7) were purchased from Procell (Wuhan, China), and maintained in DMEM supplemented with 10% FBS, penicillin (100 U/mL), and streptomycin (100 μg/mL). Human neuroblastoma cells (SH-SY5Y) were obtained from FuHeng Cell Center (Shanghai, China), cultured in DMEM/F12 supplemented with 15% FBS, penicillin (100 U/mL), and streptomycin (100 μg/ mL) in DMEM/F12. These cells were placed in an incubator at 37°C, 5% CO_2, and 95% relative humidity. Old medium was replaced with fresh medium every 24 h.

Laboratory animals

C57BL/6J male mice (aged 6–8 weeks, weighing 25–30 g) were purchased from Beijing Vital River Laboratory Animal Technology Co., Ltd. (Beijing, China). These mice were raised in a specific pathogen-free environment at 25 ± 2°C and 55 ± 5%

relative humidity. The mice were maintained on a 12-h light/dark cycle and received food and water *ad libitum*. All procedures involving experimental animals complied with the relevant guidelines provided by the Animal Ethics Committee of Guangzhou University of Chinese Medicine (NO. 20200529002).

Preparation and characterization of Cur-NCs

Cur-NCs were prepared using the anti-solvent precipitation method.[1] Cur powder was dissolved in acetone to prepare an organic phase with a concentration of 20 mg/mL. An aqueous phase was prepared with PVP K90 with a chosen concentration of 0.8 mg/mL. The organic phase was rapidly injected into the aqueous phase at a volume ratio of 1:50 and stirred at 1000 rpm/min to obtain Cur-NCs. The thermal properties of the Cur-NCs were measured using DSC (Netzsch DSC 200 F3), and the crystalline state of the Cur-NCs was analyzed using XRD at room temperature with a Brucker D8 X-ray diffractometer.

Molecular dynamics simulation

The PVP polymer (20 units per polymer block) and Cur molecular topology files were built based on the gaff force field, and the above system was placed in a cube box using GMX editconf. All molecular dynamics simulations were carried out using GROMACS 2018.4 software; the Amer99SB force field was selected, and the TIP3P model was used for water molecules. The Verlet leapfrog algorithm was used to solve the Newton equation of motion, and the integration step was set to 2 fs. In the calculation process, the van der Waals force was calculated based on the Lennard Jones function, and the non-bond truncation distance was set to 1.2 nm. The bond length of all atoms was constrained by the LinCS algorithm, and the long-range electrostatic interaction was calculated using the particle mesh Ewald method. The lattice width was set to 0.16 nm. Periodic boundary conditions were used in the simulation, and the molecular dynamics simulation period was 50 ns.

RBCm derivation

RBCms were collected based on a modified version of a previously reported method.[2] Briefly, fresh heparinized whole blood was collected from C57BL/6 mice and then centrifuged at 800 ×*g* at 4°C for 20 min to remove the plasma and buffy coat. The RBCs obtained were washed thrice with 1× PBS and suspended for 1 h in 0.25× PBS in an ice bath (inverted every 10 min). Subsequently, the cracked RBCs were centrifuged at 9000 ×g at 4°C for 10 min to remove hemoglobin. Further, a hypotonic 0.25× PBS solution (over 4 times the volume) was added, and the RBCm was washed repeatedly until the supernatant was colorless. The collected pink RBCm was purified and stored in 1× PBS.

Synthesis of DSPE-PEG$_{2000}$-RVG29

DSPE-PEG$_{2000}$-MAL (200 mg) was dissolved in 5 mL N,N-dimethylformamide (DMF). Then, RVG29 polypeptide was added and dissolved completely, and the mixture was stirred at room temperature for 12 h. Then, the reaction solution was transferred to a dialysis bag (retaining molecular weight of 3500 kDa), dialyzed, and purified in pure water for 24 h. The dialysate was collected and further freeze-dried to obtain DSPE-PEG$_{2000}$-RVG29. The synthesis of DSPE-PEG$_{2000}$-RVG29 was verified using ^{1}H-NMR spectroscopy.

Preparation of RVG29-RBCm/Cur-NCs

The RBCms derived from 400 μL of blood were incubated with 100 μg/mL of DSPE-PEG$_{2000}$-RVG29 (dispersed in PBS) in a 37°C water bath with shaking (4 h) to generate RVG29-inserted RBCm. To identify the optimal ratio of RBCm to Cur-NCs, the volume of RBCms derived from 400 μL of blood was set as 1 and then mixed with 10, 8, 6, and 4 mL of Cur-NCs separately. The RBCm and Cur-NCs were sonicated at 60 kHz for 8 min and then extruded through 400-nm, 200-nm, and 100-nm polycarbonate membranes 11 times using a lipid extruder to obtain RBCm/Cur-NCs. The mixtures with different RBCm/Cur-NCs ratios were then incubated with 20%

FBS at 37°C for 3 h. The aggregation of particles was evaluated by measuring the absorbance at 560 nm. The zeta potential and particle size of RVG29-RBCm/Cur-NCs were obtained using a particle sizing system (PSS NICOMP 380ZLS, USA), and the morphology was observed using transmission electron microscopy (TEM).

***In vitro* release**

Dialysis was employed to assess the *in vitro* release of Cur. Briefly, 1 mL of different Cur formulations was added to the dialysis bag (MWCO: 3500 kDa), which was then immersed in 10 mL of release external solution (PBS buffer, pH = 7.4, containing 0.5% Tween 80, v/v). This apparatus was placed in a 37 ± 0.5°C water bath under horizontal shaking at 100 rpm. The external solution was collected at 0, 1, 2, 4, 8, 10, 12, 24, and 48 h, and then supplemented with an equal volume of fresh medium. The Cur content was detected using a UV spectrophotometer at 427 nm.

Identification of membrane protein retention

SDS-PAGE was employed to analyze the proteins present on the prepared nanoparticles. RBCm, RVG29-RBCm, RBCm/Cur-NCs, and RVG29-RBCm/Cur-NCs were individually added to 4 times the volume of RIPA lysis buffer, shaken, and lysed for 30 min in a 4°C ice bath. The supernatant was collected after centrifugation at 10000 rpm. Then, 30 μg protein was added to each well of a 10% SDS gel and electrophoresis was performed at 100 V for 2 h. Finally, the gel was stained with Coomassie blue for 1 h and observed.

***In vitro* BBB model establishment and evaluation of NC transport**

First, bEnd.3 cells were seeded into the upper chamber of a Transwell insert at a density of 1×10^5 cells/cm^2 and cultured continuously for 5–7 days. BBB permeability was evaluated *in vitro* when the TEER value reached above 200 Ωcm^2. To evaluate the transportation of Cur from the apical to the basolateral side, equal volumes of 20 μM Cur (physical mixture of Cur and PVP K90), Cur-NCs, RBCm/Cur-NCs, and RVG29-RBCm/Cur-NCs solutions were added to the apical side (upper chamber).

Then, 200-µL samples were collected from the lower chamber at 0.5, 1, 2, 3, and 4 h after the start of the incubation period and replaced with the same volume of fresh Hank's buffer. The Cur concentration was detected using a UV-Vis spectrometer, and the apparent permeability coefficient (P_{app}) was calculated as follows:

$$P_{app} = \frac{dC / dt \times V}{C_0},$$

where V is the volume (mL) of Hank's buffer in the receptor chamber, dC/dt is the amount of Cur permeating over time (ng/mL·s), A is the surface area (cm^2) of the filter, and C_0 is the initial concentration (ng/mL) on the apical side.

Cytotoxicity assay

The *in vitro* cytotoxicity of Cur (physical mixture of Cur and PVP K90), Cur-NCs, RBCm/Cur-NCs, and RVG29-RBCm/Cur-NCs was measured using the MTT assay. RAW264.7, bEnd.3, and SH-SY5Y cells were individually seeded into 96-well plates and incubated for 24 h. The medium was then replaced with Cur formulations of different concentrations, and further incubation was performed. The cell viability was determined based on the absorbance at 570 nm.

$$\text{Cell viability (\%)} = \text{OD (sample)/OD (control)} \times 100\%.$$

Immune evasion

RAW264.7 (8×10^4 cells/well) and bEnd.3 cells (1×10^5 cells/well) were individually seeded into 12-well plates containing glass-bottom dishes and incubated for 24 h. Subsequently, the cells were treated with Cur formulations of different concentrations. After cell fixation and DAPI staining, the uptake behavior of bEnd.3 and RAW264.7 cells was observed using CLSM.

Mechanism underlying the cellular uptake of RVG29-RBCm/Cur-NCs in bEnd.3 Cells

To understand the mechanism via which bEnd.3 cells take up RVG29-RBCm/Cur-NCs, the cells were pre-treated with inhibitors of specific

endocytosis pathway (Table S1). Briefly, bEnd.3 cells were plated in 12-well plates at a density of 1 × 10^5 cells/well and incubated for 24 h. Subsequently, the cells were incubated with methyl-β-cyclodextrin (MβCD, 10 mM), chlorpromazine (CPZ, 30 μM), 5-(N-ethyl-N-isopropyl)-amiloride (EIPA, 40 μM), or the RVG29 peptide (200 μg/mL) at 37°C for 30 min. RVG29-RBCm/Cur-NCs (final Cur concentration of 100 μM) were then added and the cells were incubated for another 2 h. Cells were fixed and stained with DAPI, and the fluorescence intensity of bEnd.3 cells was imaged via CLSM.

Intracellular trafficking

The localization of Cur formulations in bEnd.3 cells was observed using trackers for subcellular organelles, including lysosomal (Lyso), mitochondrial (Mito), and endoplasmic reticulum (ER) trackers. Cells were initially plated onto confocal dishes and cultured for 24 h. They were then incubated with the Lyso Tracker (1:10000 dilution), ER Tracker (1:1000 dilution), and Mito Tracker (1:3000 dilution) for 2 h, 30 min, and 30 min, respectively. Subsequently, the medium was replaced with Cur formulations (final Cur concentration, 100 μM) for 2 h. Cells were then fixed and stained with DAPI, and the intracellular localization was observed using CLSM.

***In vitro* neuroprotective effect**

SH-SY5Y cells were initially plated at a density of 8 × 10^3/well in 96-well plates. After 24 h, the cells were pre-treated with different concentrations (1 μM, 5 μM, and 10 μM) of Cur, Cur-NCs, RBCm/Cur-NCs, and RVG29-RBCm/Cur-NCs for 2 h. Then, cells were incubated with MPP^+ (final concentration, 2 mM) for 24 h. Cell viability was measured using an MTT assay. Live/dead cell imaging was performed using calcein-AM (5 μg/mL) and PI (10 μg/mL) staining after different treatments.

Flow cytometry analysis

SH-SY5Y cells were plated at a density of 1.2 × 10^5/well in 12-well plates. After 24 h, the cells were pre-treated with 10 μM of Cur, Cur-NCs, RBCm/Cur-NCs, and

RVG29-RBCm/Cur-NCs for 2 h. Then, MPP^+ was added (final concentration, 2 mM) and the cells were incubated for 24 h. The cells were harvested and stained with Annexin V-FITC/PI for analyzing cell apoptosis. To detect intracellular ROS, the cells were treated with 10 μM 2',7'-dichlorodihydrofluorescein diacetate (DCFH-DA) and quantified using flow cytometry.

Detection of mitochondrial membrane potential

SH-SY5Y cells were treated with Cur formulations and MPP^+ as described above. Then, the cells were collected and stained using the JC-1 method. The red fluorescence of JC-1 aggregates (Ex/Em: 585/590 nm) and green fluorescence of JC-1 monomers (Ex/Em: 514/529 nm) were observed under a fluorescence microscope.

Immunofluorescence assay for α-syn

SH-SY5Y cells were first plated at a density of 1.2×10^5/well in 12-well plates. After 24 h, the cells were fixed and treated with a blocking solution (containing 0.25% Triton X-100 and 5% BSA) for 30 min. Next, the cells were incubated with a primary antibody against α-syn (1:100, Abcam) at 4°C overnight. Subsequently, these cells were incubated with a secondary antibody (1:500, Abcam) at room temperature for 2 h. At the end of this incubation, the cells were stained with DAPI, and α-syn staining was observed using CLSM.

Hemolysis test

Briefly, 150 μL of a purified 4% red blood cell suspension derived from C57BL/6J mice was incubated with 150 μL of Cur-NCs, RBCm/Cur-NCs, or RVG29-RBCm/Cur-NCs (40, 60, 80, or 100 μg/mL Cur) for 3 h at 37°C. Red blood cells suspended in 0.9% NaCl and water were set as the negative and positive controls, respectively. Samples were then centrifuged at 8000 ×g, and the absorbance of the supernatants was measured at 570 nm via a UV–vis spectrophotometer. Hemolysis rates were quantified as follows:

$$\text{Hemolysis (\%)} = \frac{A_{\text{Sample}} - A_{\text{Negative Control}}}{A_{\text{Positive Control}} - A_{\text{Negative Control}}} \times 100\%$$

Immunogenic response and neuroinflammation analysis

Healthy male C57BL/6J mice were injected with Cur-NCs, RBCm/Cur-NCs, and RVG29-RBCm/Cur-NCs at the dose of 2 mg/kg Cur, and the mice in the control group received the same volume of saline. After 8 rounds of administration on every alternate day during a 15-day period, the serum, midbrain, and striatum tissues were collected for further analyses. The levels of cytokines such as TNF-α, IFN-γ, IL-4, and IL-6; IgM; IgG; IgA; and complement C3 and C4 were detected in the serum using ELISA. The transcriptional levels of IL-1β, TNF-α, and IL-6 in the midbrain and striatum tissues were quantified using qRT-PCR.[3]

Pharmacokinetic studies

Healthy C57BL/6J mice were treated with different Cur formulations (2 mg Cur/kg) via tail vein injections. Plasma and brain samples were collected from treated mice after perfusion at 0.25, 0.5, 1, 2, 4, 8, 12, 24, 48, and 72 h after treatment. Subsequently, LC-MS/MS was performed to measure the Cur concentration in all samples. Pharmacokinetic parameters were further estimated using DAS 2.0 software. These parameters included the peak concentration of Cur (C_{max}), the elimination half-life ($T_{1/2}$), area under the curve for Cur levels in the plasma/brain from time zero to t ($AUC_{0\text{-}t}$), and the mean residence time ($MRT_{0\text{-}t}$).

***In vivo* imaging**

To evaluate the brain-targeting ability of the biomimetic NCs, the prepared nano-formulations were labeled with Cy5 via lipid insertion. Cy5, Cy5@RBCm/Cur-NCs, and Cy5@RVG29-RBCm/Cur-NCs were separately injected into the tail vein of healthy mice, and fluorescence signals were recorded from major organs (brain, heart, liver, spleen, lungs, and kidneys) at different time-points using a live imaging system (Berthold LB983). Fluorescence signals were then quantified

using IndiGo software.

Development of the PD mouse model and treatment protocols

C57BL/6J male mice were randomly divided into 7 groups (*n* = 8): ① control group, ② MPTP group, ③ L-DOPA group, ④ Cur group, ⑤ Cur-NCs group, ⑥ RBCm/Cur-NCs group, and ⑦ RVG29-RBCm/Cur-NCs group. MPTP·HCl was dissolved in a 0.9% NaCl solution, and mice were intraperitoneally injected with MPTP at a dose of 25 mg/kg for 5 consecutive days.[4] L-DOPA was suspended in saline and administered intraperitoneally at a dosage of 25 mg/kg. An equal dose of Cur was administered in all Cur groups (2 mg/kg). Additionally, in the Cur group, free Cur was diluted in dimethylacetamide, PEG_{400}, and a 5% dextrose solution (15: 45: 40, v/v/v) according to the reported method.[5] The indicated therapeutic agents were administered once every other day for a total of 8 doses. The weight of the mice was monitored and recorded during the administration period.

Behavioral tests

For the pole test, the mice were placed in a head upward position on top of a vertical rod with a rough surface (1 cm in diameter and 50 cm in height). The mice spent on turning back (T-turn) and the total time required to climb to the bottom of the rod (T-total) were recorded.[6] For the rotarod test, the mice were placed on a rod (7 cm in diameter) rotating at a fixed speed of 30 rpm. During the 2-min test, the latency to fall and the number of drops were recorded for each animal. These experiments were repeated three times for each mouse, and analyses were performed using the average value.

For the open-field test, mice were individually placed in an empty box (60 cm × 60 cm × 40 cm), and the environment was kept dark and quiet during the experiment. Before the test, each mouse was placed in the center of the empty box and allowed to explore it freely for 10 minutes to ensure adaption. Then, the trajectory of the mice was recorded for 15 min during the test and analyzed.

For the gait dynamics test, a Digigait^{TM} Imaging system (Mouse Specifics Inc.)

was conducted as previously reported.[7] Firstly, the mice were trained to walk on the belt at a speed of 22 cm/s. Then, the gait of the mice walking continuously in the center of the visual field was recorded. The portions of the paw, gait parameters, and relevant index values were identified by the software. The definition of the parameters of interest are depicted in **Table S3**.

Quantification of TH^+ neurons

Immunofluorescence analysis was performed to quantify the number of TH^+ neurons in the SNpc according to previously described protocols.[8]. Brains harvested from mice were fixed in 4% paraformaldehyde (PFA) and then dehydrated in 30% sucrose. After dehydration, the brains were cut into 30-μm-thick sections, which were blocked with 10% goat serum for 30 min. The sections were then incubated with an anti-TH antibody (1:2000) overnight at 4°C. Subsequently, the sections were rinsed thrice in PBS and incubated with an Alexa fluor 594-conjugated secondary antibody at room temperature for 2 h. After DAPI staining, red fluorescence was observed under a fluorescence microscope, and the TH^+ cells in the SNpc were quantified using ImageJ software.

Detection of oxidative stress levels

The levels of SOD, malondialdehyde (MOD), and GSH in the striatum of mice were detected following standard protocols of Beyotime Biotechnology (Shanghai, China). The levels of glutamate dehydrogenase (GDH), ATP, ROS, and MOD in the midbrain were measured in a similar manner.

Measurement of DA levels

The striatum was isolated and weighed for further analysis. The sample was placed in 0.4 M/L $HClO_4$ (10 μL/mg tissue) and sonicated on ice. The mixture was centrifuged at 10000 rpm at 4°C for 10 min to precipitate the protein. The concentrations of dopamine and its metabolites HVA and DOPAC were determined using chromatography (ESA, Chelmsford, Ma, USA).

Western blotting

The harvested midbrain and striatum were weighed and lysed with RIPA buffer in the presence of protease/phosphatase inhibitors (ratio of tissue to lysate was 1:9, w/v). Equivalent protein samples (30 μg) were then separated on an SDS-PAGE gel (10% for TH detection, 12% for α-syn detection) and transferred onto PVDF membranes. After blocking in 5% bovine serum albumin (BSA) for 2 h, the membranes were incubated with the following primary antibodies at 4°C overnight: TH (1:5000), α-syn (1:1000), and β-actin (1:3000). The blots were rinsed thrice and then incubated with the appropriate secondary antibodies for 2 h. The signals were detected using the ECL Chemiluminescent Reagent. Protein bands were analyzed using ImageJ software with β-actin as the reference standard.

***In vivo* biocompatibility**

After the treatment period, blood was obtained from mice in each group for blood cell and biochemical index analysis. The heart, liver, spleen, lungs, and kidneys were collected and weighed to calculate the organ index according to the following formula:

$$\text{Organ Index (\%)} = \frac{\text{Weigh of organ (mg)}}{\text{Weigh of body (g)}} \times 100\%$$

Moreover, these organs were fixed in 4% PFA and cut into 5 μm-thick sections. For pathological analysis, the tissue sections were stained with H&E and potential organ damage was evaluated.

Statistical analysis

All data were expressed as the mean ± SD and statistically analyzed using GraphPad Prism 8.0 software. The statistical analyses included a student's t-test, one-way ANOVA, and two-way ANOVA. $P < 0.05$ was designated as the threshold for statistical significance.

Results

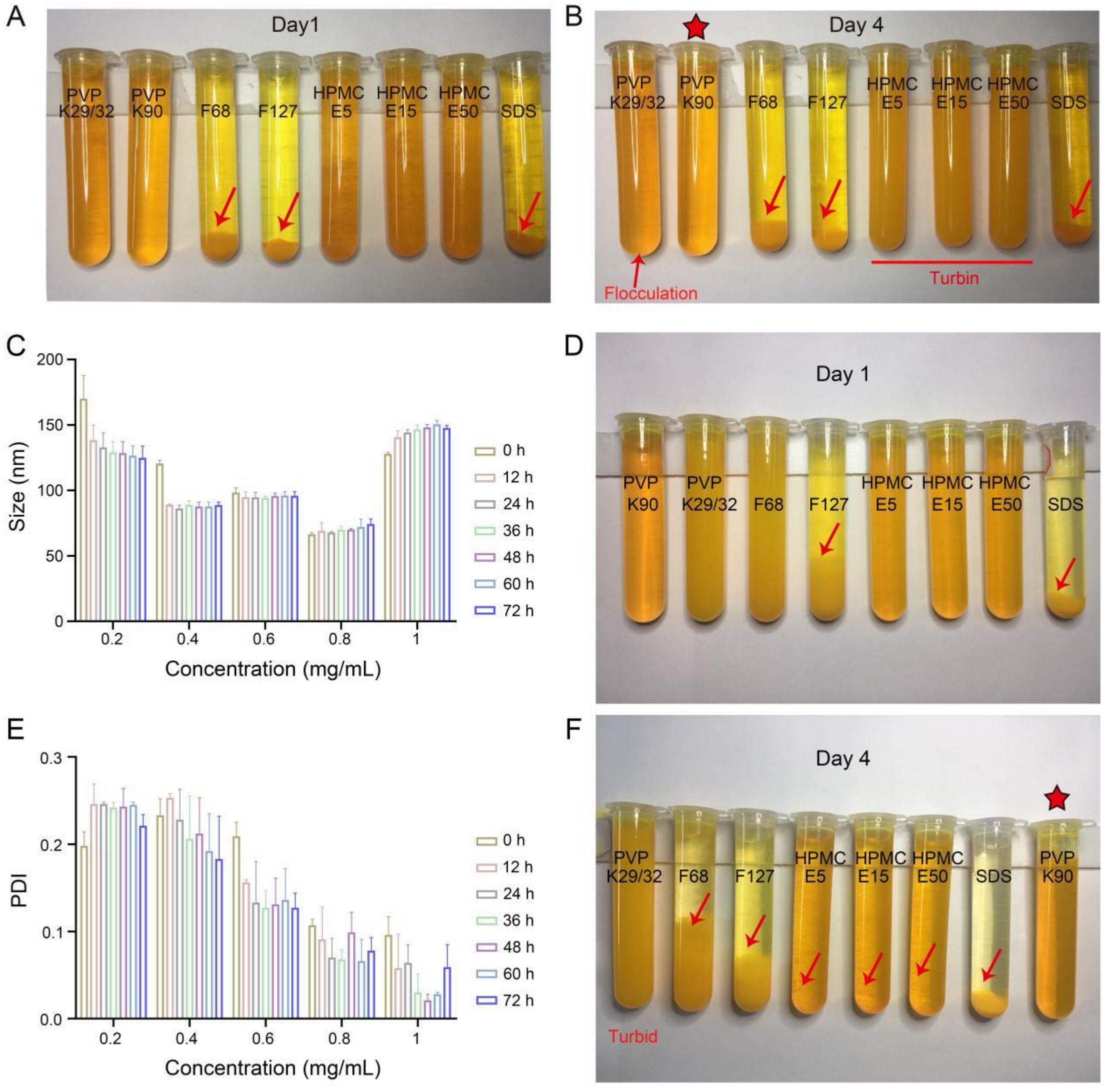

Figure S1. Preparation of Cur-NCs. **(A)** Stability of various Cur-NCs formulations at 0.2 mg/mL stabilizer concentration on day 1. At this concentration, Cur-NCs were prepared with the stablizers of pluronic F68, pluronic F127 and sodium dodecyl sulfate (SDS). **(B)** Stability of various Cur-NCs formulations at 0.2 mg/mL stabilizer concentration on day 4. Flocculation occurred in PVP K29/32, and obvious turbidity occurred in HPMC series, while Cur-NCs prepared with PVP K90 remained stable and clear. **(C)** The particle size of Cur-NCs under different concentrations of PVP K90 within 72 h. Cur-NCs prepared with 0.8 mg/mL PVP K90 showed minimum particle size. **(D)** Stability of various Cur-NCs formulations at 0.8 mg/mL stabilizer concentration on day 1. **(E)** The PDI of Cur-NCs under different concentrations of PVP K90 within 72 h. The particle size distribution of Cur-NCs prepared with 0.8

mg/mL PVP K90 were small and uniform. **(F)** Stability of various Cur-NCs at 0.8 mg/mL stabilizer concentration on day 4. Cur-NCs prepared with 0.8 mg/mL PVP K90 maintained transparent and stable.

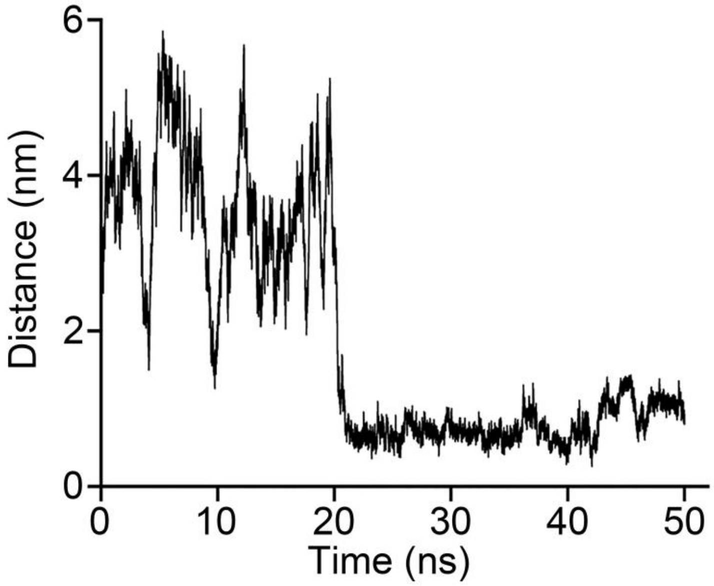

Figure S2. Time evolution of the center mass distance between Cur and PVP K90.

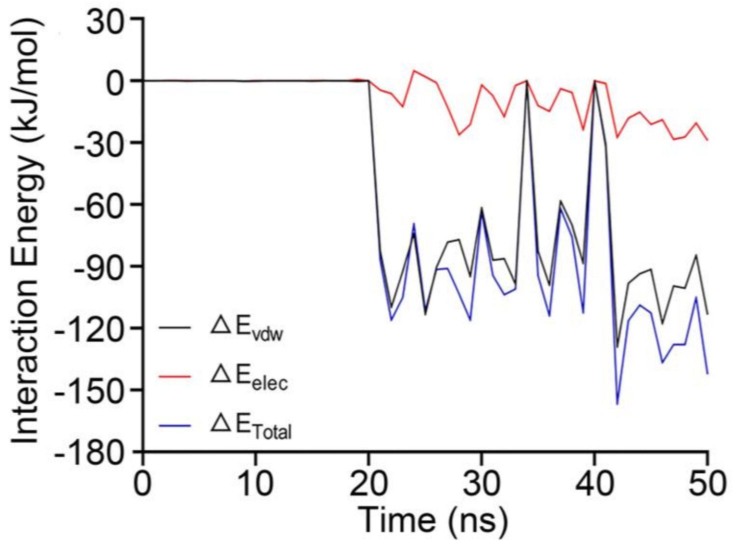

Figure S3. Time evolution of the interaction energy between Cur and PVP K90 over 50 ns.

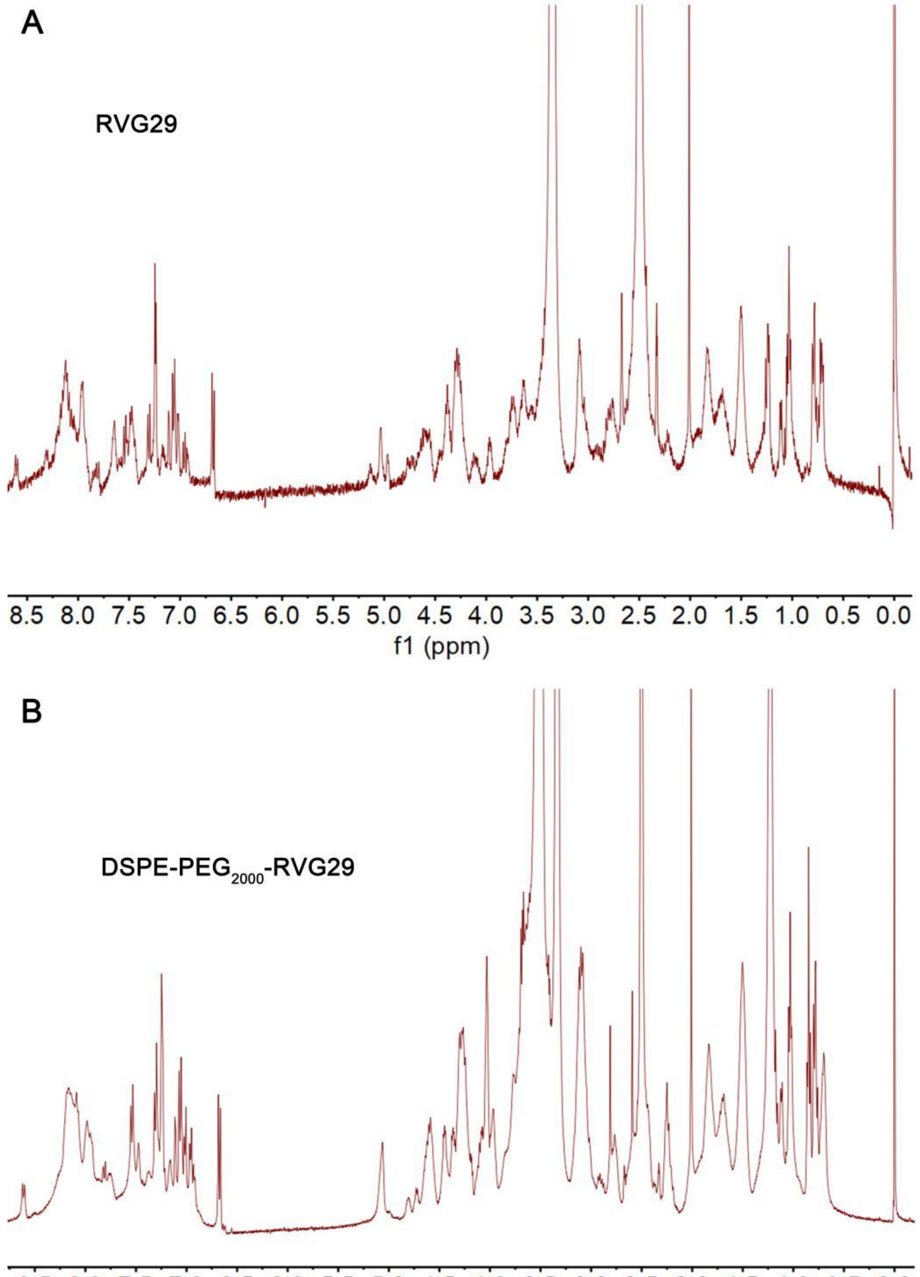

Figure S4. ^{1}H-NMR spectra of **(A)** RVG29 and **(B)** DSPE-PEG$_{2000}$-RVG29.

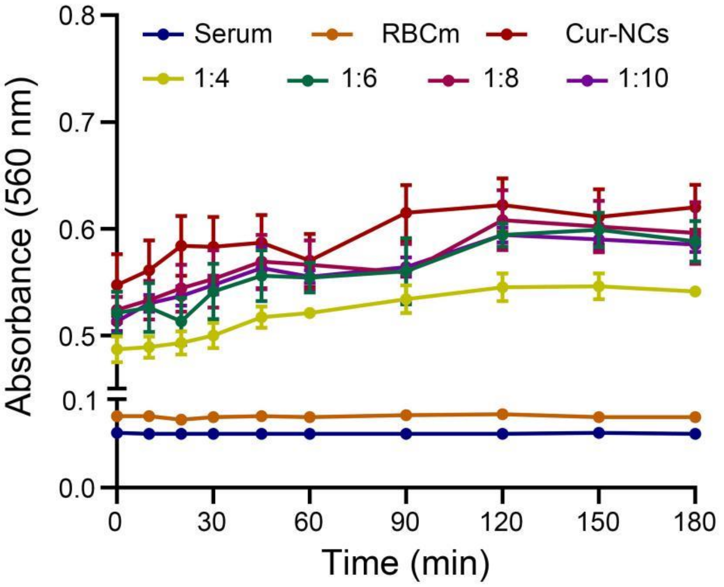

Figure S5. Optimization of Cur-NCs to RBCm ratios based on short-term serum stability study (n = 5).

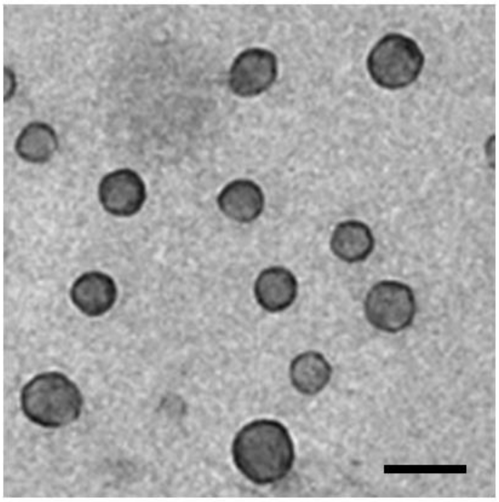

Figure S6. TEM images of RBCm/Cur-NCs. Scale bar: 100 nm.

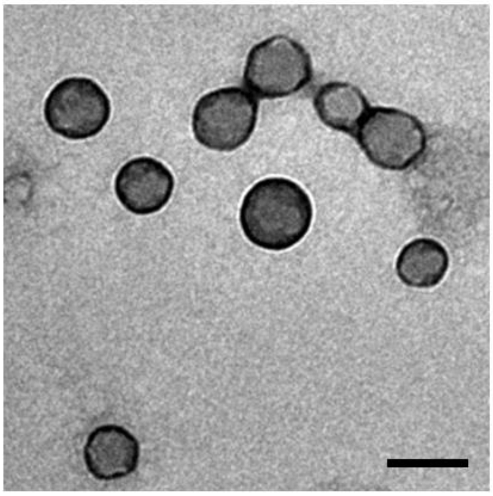

Figure S7. TEM images of RVG29-RBCm/Cur-NCs. Scale bar: 100 nm.

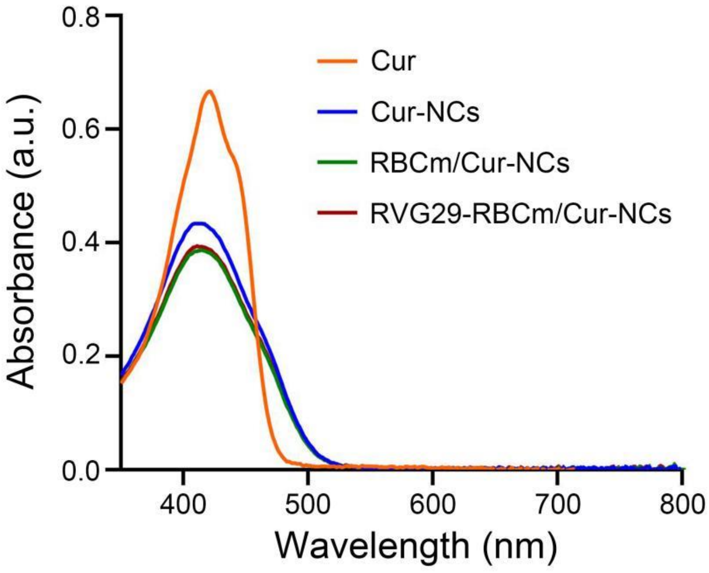

Figure S8. UV–Vis absorption spectra of different Cur formulations.

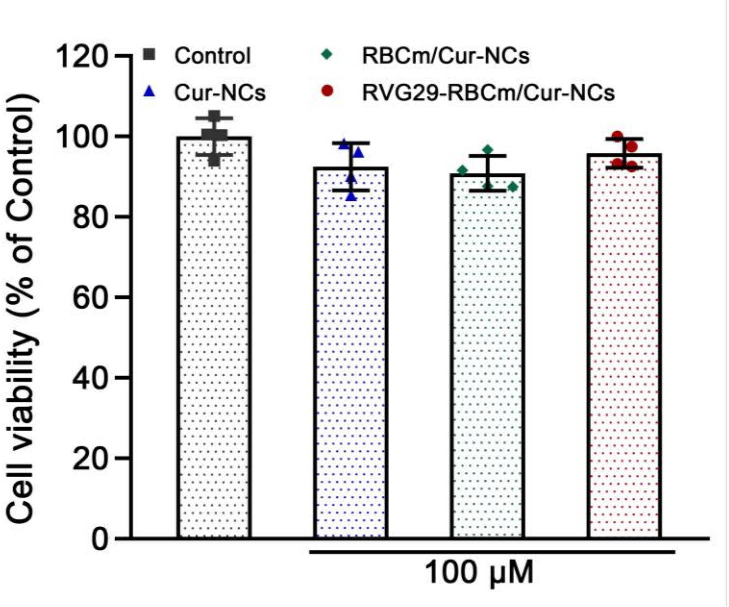

Figure S9. Cytotoxicity evaluation of different Cur formulations co-incubated with RAW264.7 cells for 2 h (*n*=4).

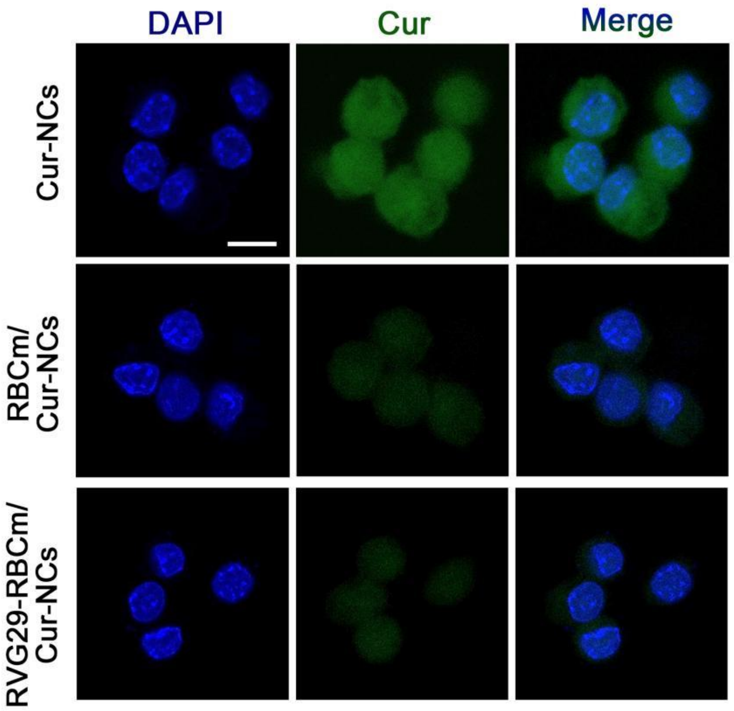

Figure S10. CLSM images of RAW264.7 cells after incubation with various Cur formulations. Scale bar: 10 μm.

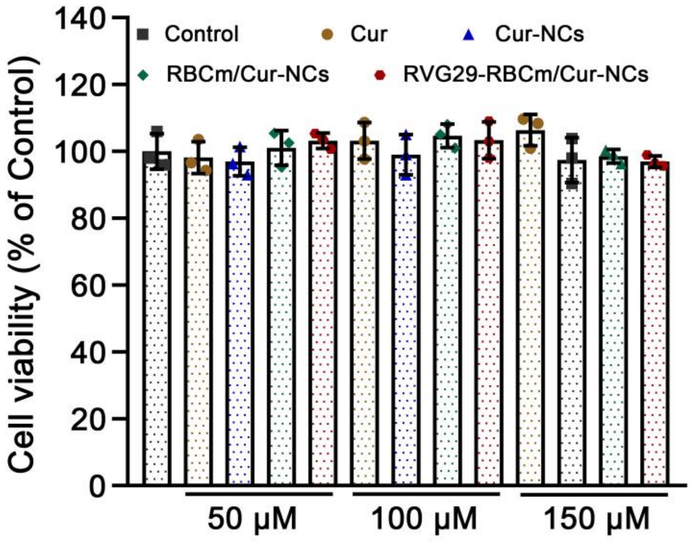

Figure S11. Cytotooxicity evaluation of different Cur formulations co-incubated with bEnd.3 cells for 4 h (*n*=3).

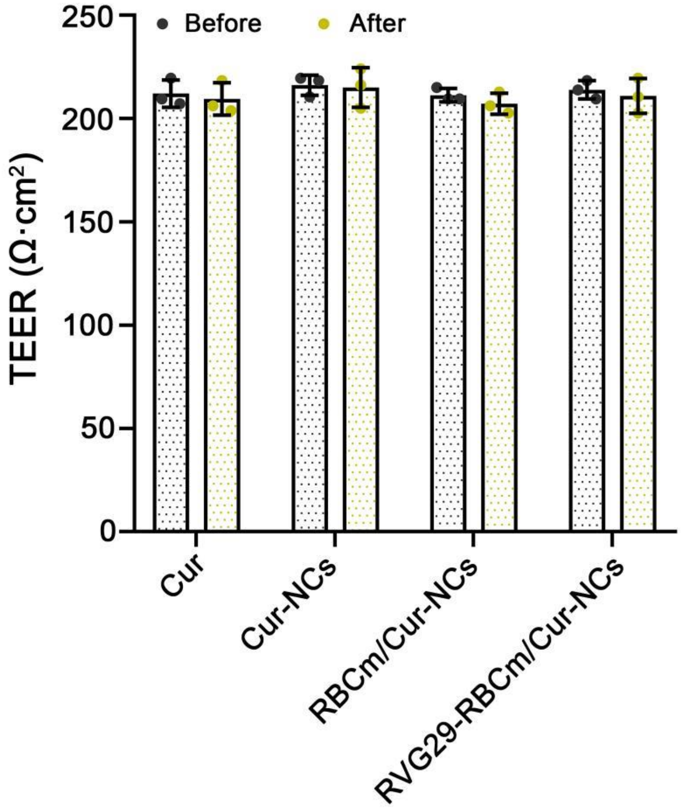

Figure S12. The TEER values of bEnd.3 cell monolayer before and after transport (*n* = 3).

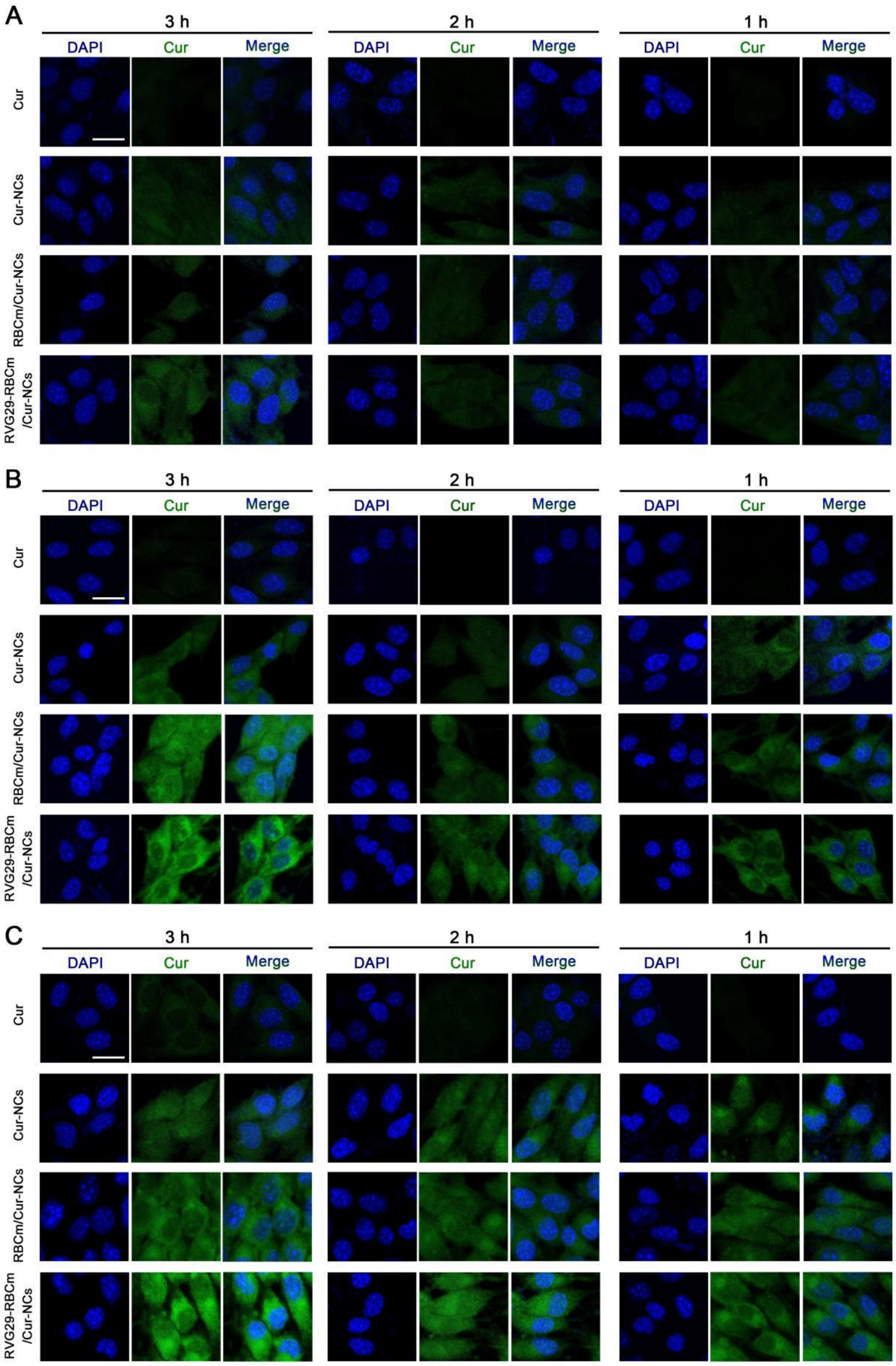

Figure S13. Cellular uptake of various Cur formulations in bEnd.3 cells at different Cur concentrations of 50 μM (**A**), 100 μM (**B**) and 150 μM (**C**). Scale bar: 20 μm

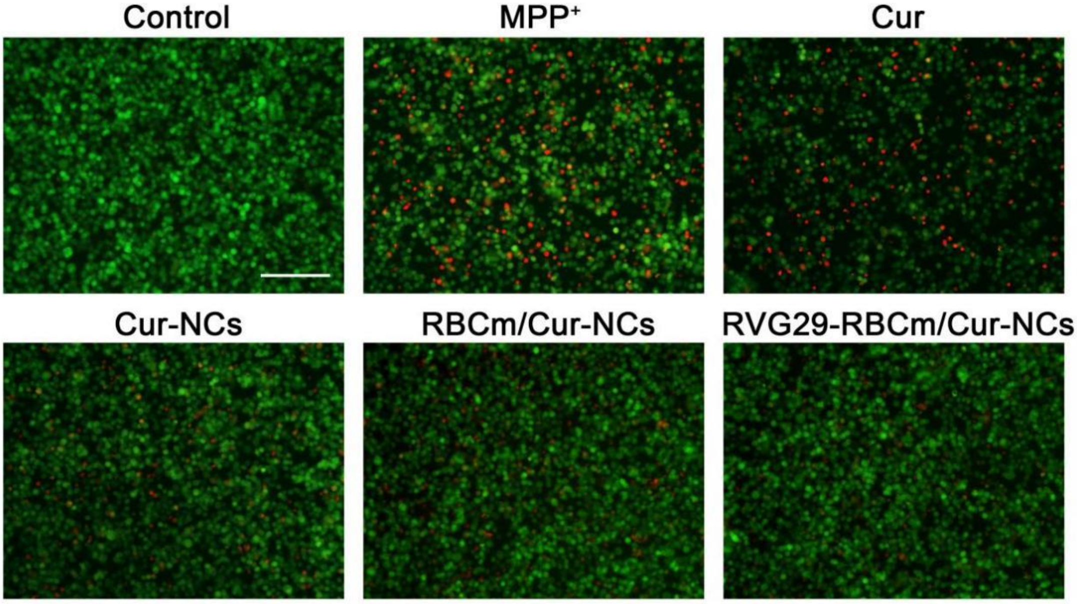

Figure S14. Live/dead staining of different Cur formulations against MPP^+-induced cell death and cytotoxicity. Scale bar: 250 μm.

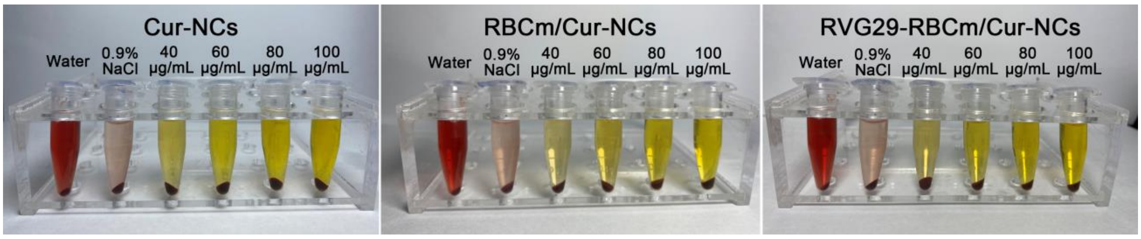

Figure S15. Hemolysis study of Cur-NCs, RBCm/Cur-NCs and RVG29-RBCm/Cur-NCs.

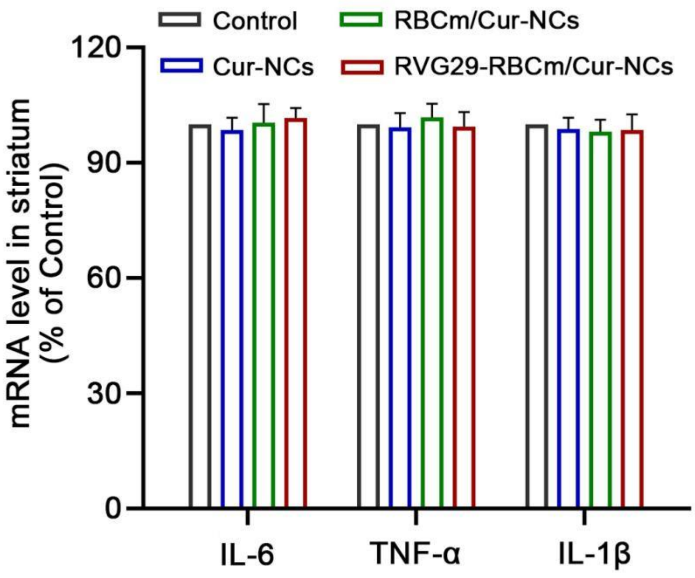

Figure S16. Levels of inflammatory factors in striatum.

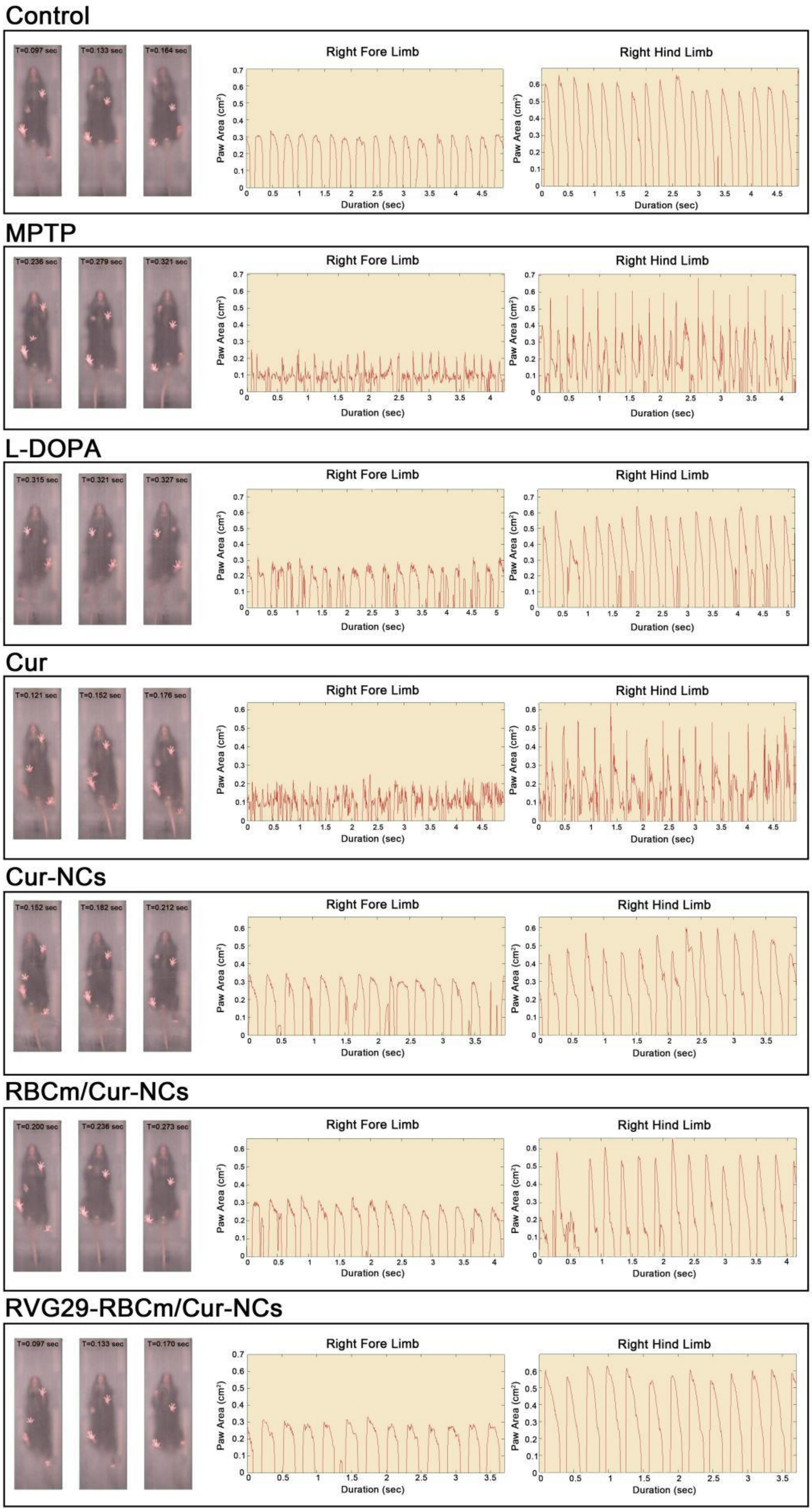

Figure S17. Representative images depicting pattern gait signal of right limbs of mice after different treatments.

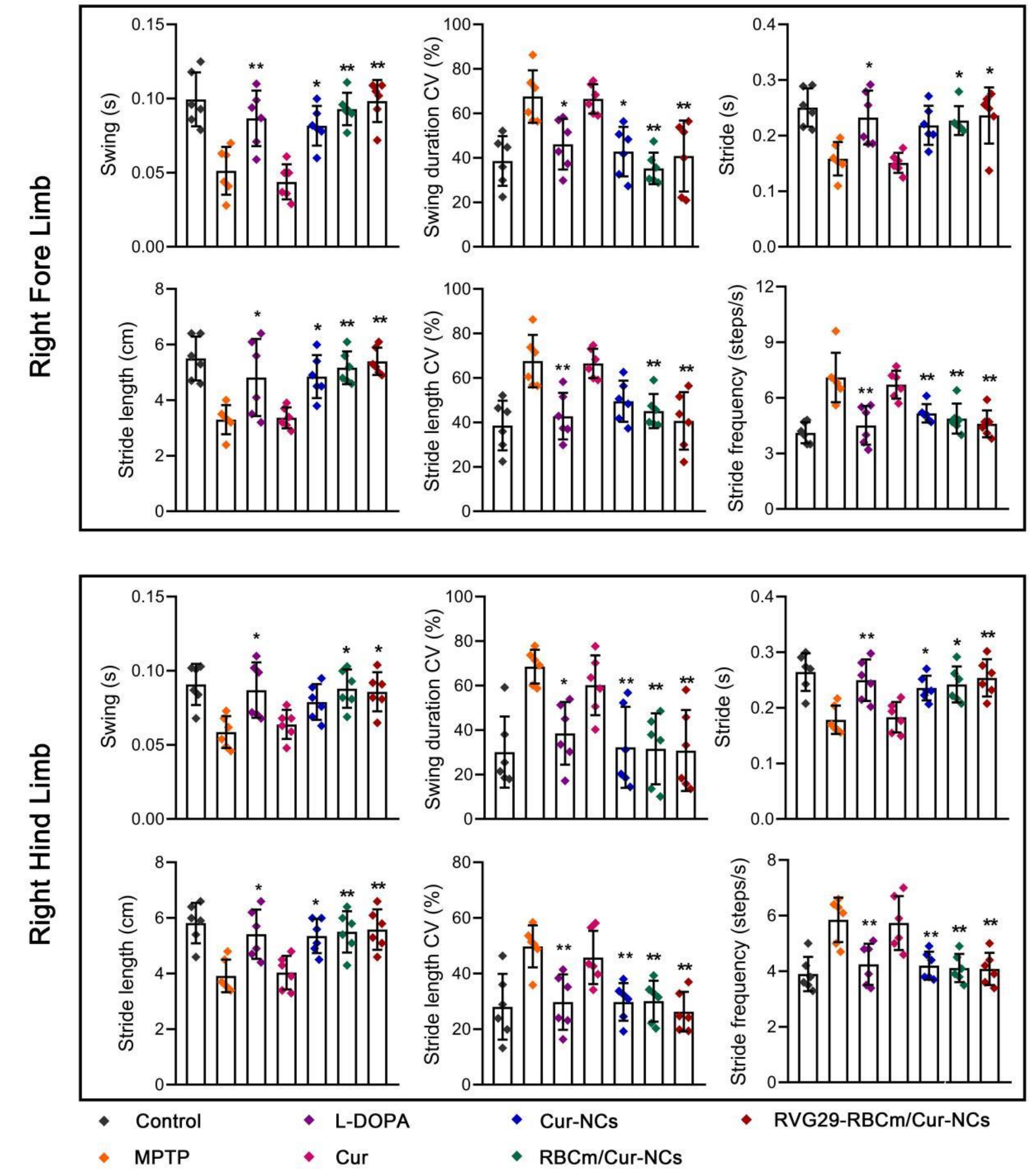

Figure S18. Gait parameters including swing, swing duration CV, stride, stride length, stride length CV and stride frequency of right fore limb and right hind limb from the mice after different treatments. $^{*}P < 0.05$ and $^{**}P < 0.01$ *vs* the MPTP group.

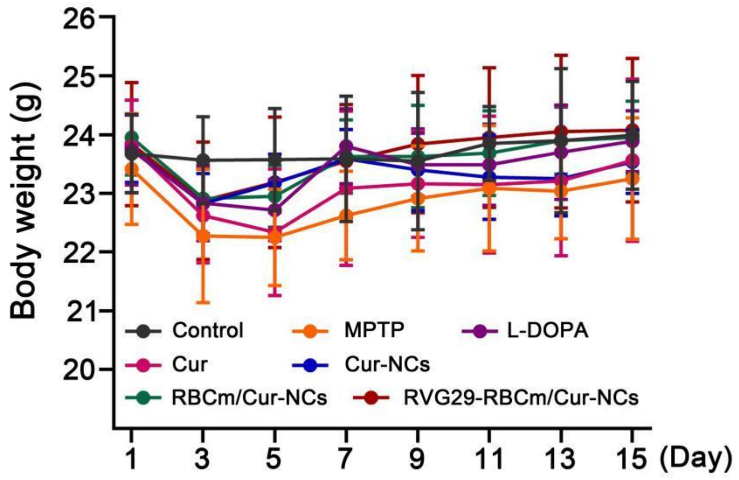

Figure S19. Body weight curve of mice ($n = 8$).

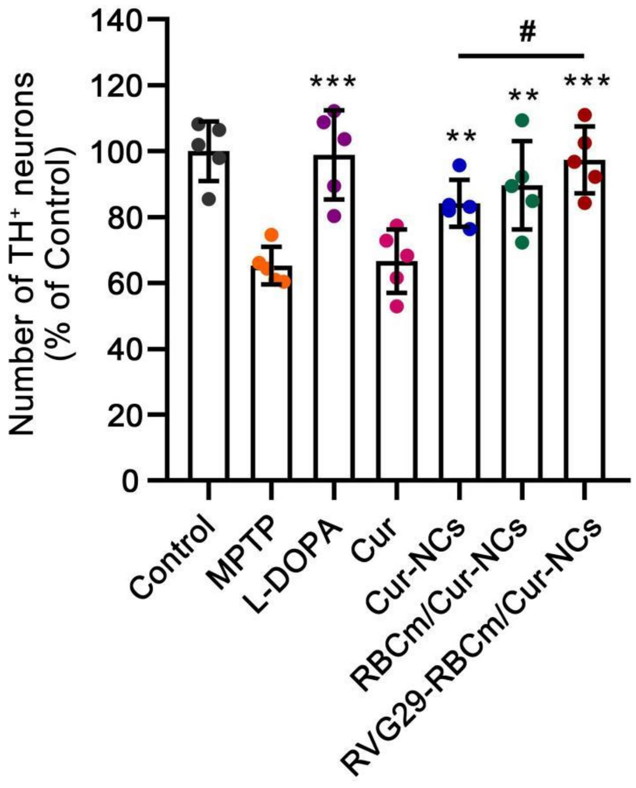

Figure S20. The number of TH^{+} neurons in SNpc ($n = 5$). $^{*}P < 0.05$, $^{**}P < 0.01$ and $^{***}P < 0.001$ *vs* the MPTP group. $^{\#}P < 0.05$ with respect to the RVG29-RBCm/Cur-NCs group.

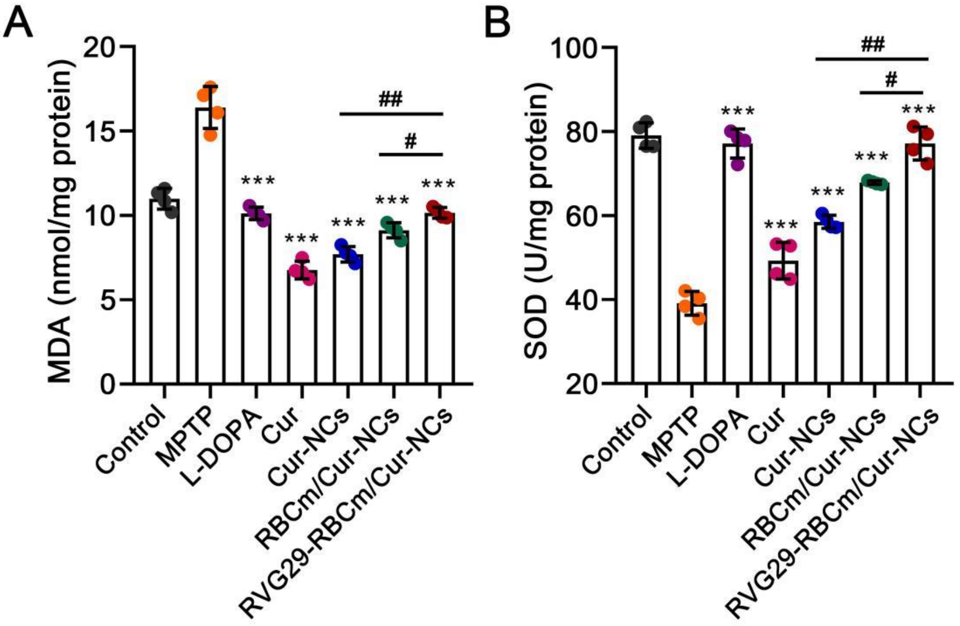

Figure S21. The contents of **(A)** MDA and **(B)** SOD in striatum. $^{*}P < 0.05$ and $^{**}P < 0.01$ *vs* the MPTP group. $^{\#}P < 0.05$ and $^{\#\#}P < 0.01$ *vs* the RVG29-RBCm/Cur-NCs group. MDA: malondialdehyde, SOD: superoxide dismutase.

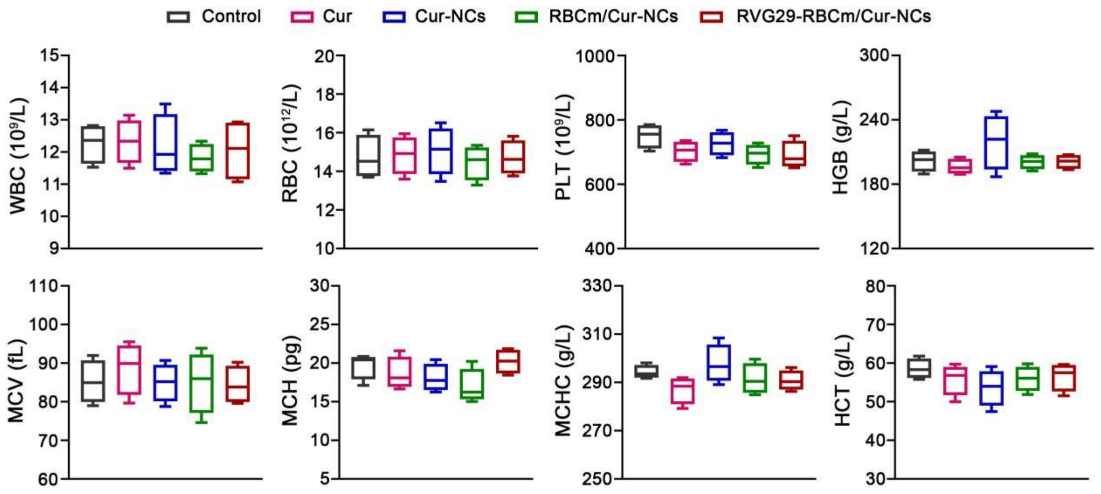

Figure S22. Blood routine analysis (n = 4). WBC: white blood cells, RBC: red blood cells, PLT: platelets, HGB: hemoglobin, MCV: mean corpuscular volume, MCH: mean corpuscular hemoglobin, MCHC: mean corpuscular hemoglobin concentration, HCT: hematocrit.

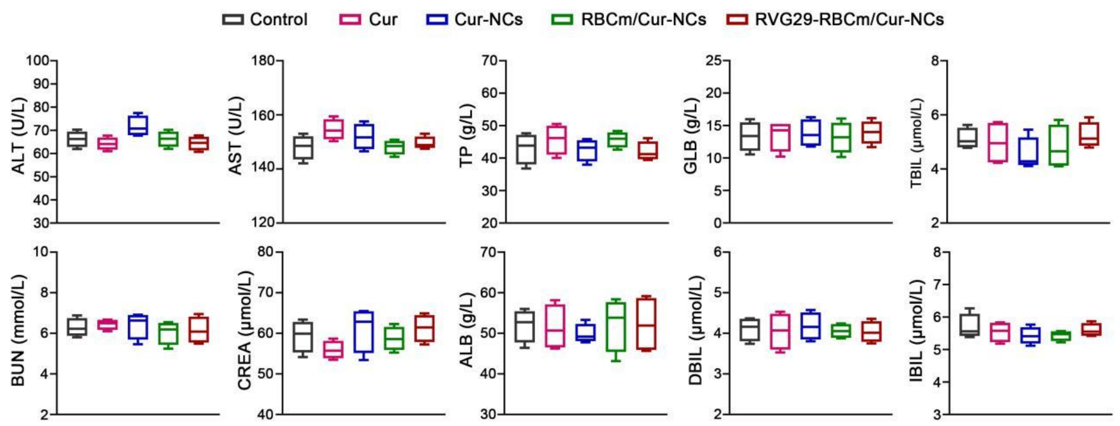

Figure S23. Biochemical analysis of blood (n = 4). ALT: alanine aminotransferase, AST: aspartate aminotransferase, TP: total protein, GLB: globulin, TBIL: total bilirubin test, BUN: blood urea nitrogen, CREA: creatinine, ALB: albumin, DBIL: direct bilirubin, IBIL: indirect bilirubin.

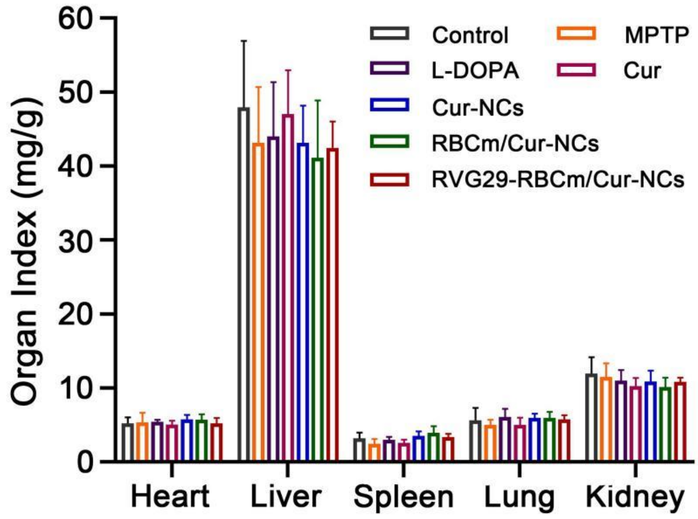

Figure S24. Organ index of mice (n = 8).

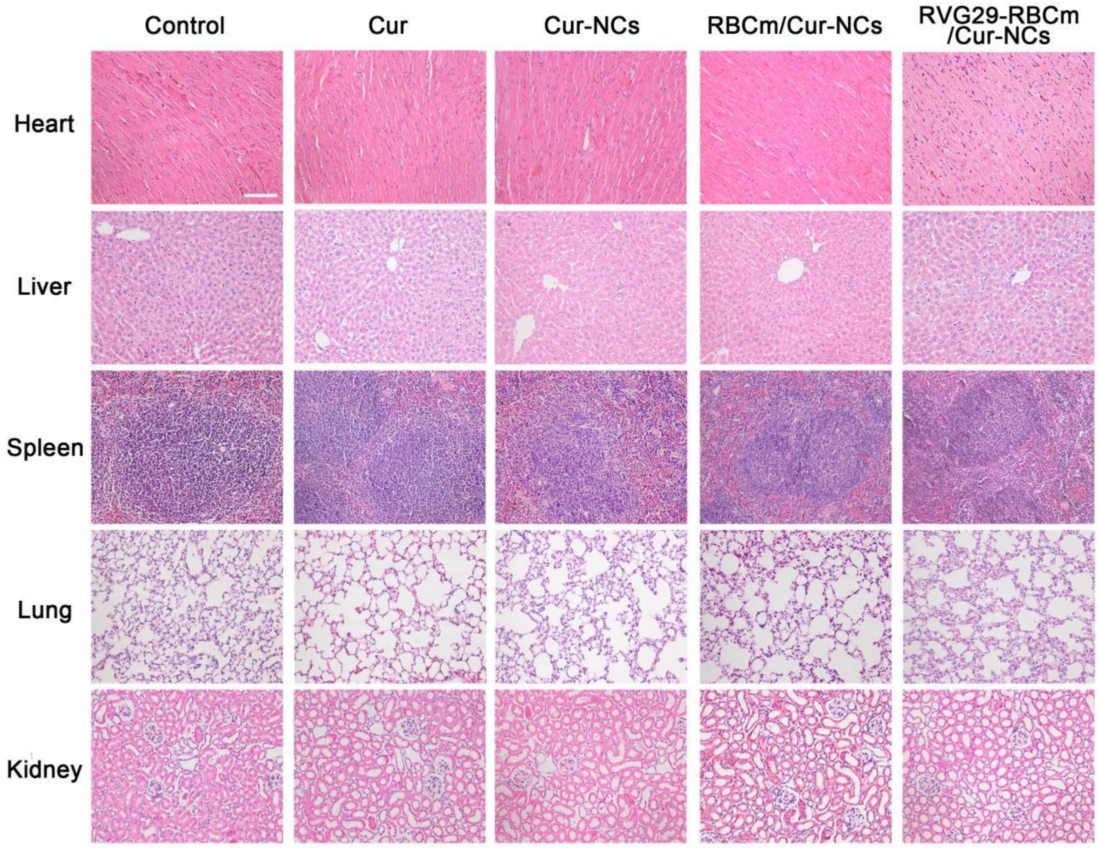

Figure S25. H&E staining of main organs in mice. Scale bar: 100 μm.

Table S1. Inhibitors used for exploring uptake mechanism of RVG29-RBCm/Cur-NCs.

Inhibitors	Target pathway	Property	Function	Working concentration
Methyl-β-cyclodextrin (MβCD)	Lipid- and caveolae-dependent endocytosis.	Depletes cholesterol.	Removes cholesterol from plasma membrane.	10 mM
Chlorpromazine (CPZ)	Clathrin-dependent endocytosis.	Cationic amphiphilic compound.	Disrupts AP2 and clathrin complex formation at the cell surface.	30 μM
5-(N-ethyl-N-isopropyl)-amiloride (EIPA)	Macropincytosis.	Blocks Na^+/H^+ion channels.	Inhibits Na^+/H^+ exchange and thereby disrupts macropinocytosis.	40 μM
RVG29	nAChR receptor-mediated specific uptake.	Binding to nAChR receptors.	Pre-supersaturated nAChR receptors and thereby inhibits specific uptake.	200 μg/mL

Table S2. Pharmacokinetic parameters in the plasma and brain (n = 4).

Parameters	Cur	RBCm/Cur-NCs	RVG29-RBCm/Cur-NCs
Plasma			
$T_{1/2}$ (h)	0.448 ± 0.036	15.984 ± 1.285*	20.036 ± 1.573$^{**\#}$
T_{max} (h)	0.500 ± 0	0.500 ± 0	0.500 ± 0
C_{max} (μg/mL)	0.106 ± 0.087	0.658 ± 0.052**	0.596 ± 0.045$^{**\#}$
$AUC_{0\text{-}t}$ (μg·h/mL)	0.177 ± 0.013	10.271 ± 0.918**	10.186 ± 0.904**
$MRT_{0\text{-}t}$ (h)	0.864 ± 0.074	18.470 ± 1.547**	21.252 ± 1.892$^{**\#\#}$
F	100%	5802.82%	5754.80%
Brain			
$T_{1/2}$ (h)	2.575 ± 0.202	12.849 ± 1.115**	15.236 ± 1.340$^{**\#\#}$
T_{max} (h)	0.875 ± 0.250	4.500 ± 1.000*	7.500 ± 1.000$^{*\#}$
C_{max} (μg/g)	0.021 ± 0.004	0.072 ± 0.009*	0.125 ± 0.015$^{**\#}$
$AUC_{0\text{-}t}$ (μg.h/g)	0.068 ± 0.008	1.744 ± 0.151**	3.550 ± 0.332$^{**\#}$
$MRT_{0\text{-}t}$ (h)	2.259 ± 0.219	20.076 ± 1.896**	21.841 ± 1.777$^{**\#}$

$^{*}p<0.05$ and $^{**}p<0.01$ *vs* the Cur group. $^{\#}p < 0.05$ and $^{\#\#}p < 0.01$ *vs* the RBCm/Cur-NCs group.

Table S3. Definitions of gait parameters.

Parameters	Units	Definition
Swing	s	Time duration of the swing phase (no paw contact with belt).
Swing Duration CV	CV%	CV was calculated from the equation: 100 × standard deviation/mean value (the variability normalized to the mean)
Stride	s	Time duration of one complete stride for one paw. Equal to the sum of stance duration and swing duration.
Stride Length	cm	The spatial length that a paw traverses through a given stride.
Stride Length CV	CV%	Coefficient of variation in stride length. CV was calculated from the equation: 100 × standard deviation/mean value (the variability normalized to the mean)
Stride Frequency	Steps per second	The cadence or the number of times each second that a paw takes a complete stride.

*CV: Coefficient of variation.

References

(1) Liu, Y.; Liu, W.; Xiong, S.; Luo, J. S.; Li, Y.; Zhao, Y. Y.; Wang, Q.; Zhang, Z. X.; Chen, X. J.; Chen, T. K. Highly stabilized nanocrystals delivering Ginkgolide B in protecting against the Parkinson's disease. *Int. J. Pharm.* **2020**, *577*.

(2) Chai, Z. L.; Ran, D. N.; Lu, L. W.; Zhan, C. Y.; Ruan, H. T.; Hu, X. F.; Xie, C.; Jiang, K.; Li, J. Y.; Zhou, J. F.; et al. Ligand-Modified Cell Membrane Enables the Targeted Delivery of Drug Nanocrystals to Glioma. *ACS Nano* **2019**, *13* (5), 5591-5601.

(3) You, L.; Wang, J.; Liu, T.; Zhang, Y.; Han, X.; Wang, T.; Guo, S.; Dong, T.; Xu, J.; Anderson, G. J.; et al. Targeted Brain Delivery of Rabies Virus Glycoprotein 29-Modified Deferoxamine-Loaded Nanoparticles Reverses Functional Deficits in Parkinsonian Mice. *ACS Nano* **2018**, *12* (5), 4123-4139.

(4) Wei, Y.; Lu, M.; Mei, M.; Wang, H. R.; Han, Z. T.; Chen, M. M.; Yao, H.; Song, N. S.; Ding, X.; Ding, J. H.; et al. Pyridoxine induces glutathione synthesis via PKM2-mediated Nrf2 transactivation and confers neuroprotection. *Nat. Commun.* **2020**, *11* (1), 941.

(5) Gao, C. H.; Wang, Y. L.; Sun, J. J.; Han, Y.; Gong, W.; Li, Y.; Feng, Y.; Wang, H.; Yang, M. Y.; Li, Z. P.; et al. Neuronal mitochondria-targeted delivery of curcumin by biomimetic engineered nanosystems in Alzheimer's disease mice. *Acta Biomater.* **2020**, *108*, 285-299.

(6) Guo, M.; Yang, C.; Li, B.; Cheng, S.-X.; Guo, Q.; Ming, D.; Zheng, B. Bionic Dormant Body of Timed Wake-Up for Bacteriotherapy in Vivo. *ACS Nano* **2022**, *16* (1), 823-836.

(7) Liu, J. Y.; Liu, C.; Zhang, J. F.; Zhang, Y. M.; Liu, K. Y.; Song, J. X.; Sreenivasmurthy, S. G.; Wang, Z. Y.; Shi, Y. S.; Chu, C. C.; et al. A Self-Assembled alpha-Synuclein Nanoscavenger for Parkinson's Disease. *ACS Nano* **2020**, *14* (2), 1533-1549.

(8) Liu, Y.; Hong, H. H.; Xue, J. C.; Luo, J. S.; Liu, Q.; Chen, X. J.; Pan, Y.; Zhou, J. W.; Liu, Z. M.; Chen, T. K. Near-Infrared Radiation-Assisted Drug Delivery Nanoplatform to Realize Blood-Brain Barrier Crossing and Protection for Parkinsonian Therapy. *ACS Appl. Mater. Interfaces* **2021**, *13* (31), 37746-37760.
